# Symmetric cancer spheroid-fibroblast organization revealed in 3D by high-throughput microscopy

**DOI:** 10.1038/s42003-026-10592-3

**Published:** 2026-07-27

**Authors:** Noam Zoref, Maytal Avrashami, Nadav Opatovski, Paul Keselman, Yosi Shamay, Yoav Shechtman

**Affiliations:** 1 Faculty of Biomedical Engineering, Technion - Israel Institute of Technology, Haifa, Israel; 2 Russell Berrie Nanotechnology Institute, Technion - Israel Institute of Technology, Haifa, Israel; 3 Sartorius Stedim North America Inc., Bohemia, NY USA; 4 Faculty of Electrical and Computer Engineering, Technion - Israel Institute of Technology, Haifa, Israel

**Keywords:** Image processing, 3-D reconstruction, Wide-field fluorescence microscopy

## Abstract

High-throughput microscopy enables large-scale analysis of cancer spheroid interactions. Integrating this technique with point-spread function (PSF) engineering, a customized modification of the optical system, provides 3D imaging capabilities that enable volumetric analysis of the samples from a minimal number of images, thereby reducing phototoxicity and improving temporal resolution. However, existing methods for extracting volumetric information using PSF engineering have been demonstrated primarily on point sources or rely on supervised training data, limiting their general applicability to complex biological samples. Here, we reveal a unique symmetric spheroid-fibroblast interaction pattern using high-throughput imaging. We further characterize these interactions using a PSF-engineered high-throughput microscope, introducing unsupervised methods for 3D localization from single or dual image acquisitions. Our analysis uncovers surprising spatial cellular trajectories of fibroblasts as they approach and interact with spheroids. It further identifies a characteristic 3D distance between adjacent fibroblast clusters and enables live detection of drug-induced perturbations to these interaction patterns.

## Abstract (web summary)

Cancer spheroid-fibroblast “flower-like” interactions revealed and tracked in 3D using high-throughput imaging and PSF engineering.

## Introduction

Multicellular tumor spheroids (MCTSs) are a widely used form of three-dimensional (3D) cell culture that more accurately replicates in vivo tumor conditions compared to traditional two-dimensional (2D) cultures^1^. Their structure mimics key physiological features of solid tumors, including extracellular matrix (ECM) development, concentration gradients, hypoxia, and central necrosis^2,3^. These additional features often result in cellular behaviors not observed in 2D systems with better correlation with in vivo observations^4,5^. However, solid tumors have complex interactions with multiple cell types, such as immune, endothelial, and stromal cells, resulting in phenotypes that cannot be captured by spheroids composed of a single cancer cell line^6–8^. Therefore, co-culture spheroids composed of two or more cell types are used to better model these interactions since they provide a more realistic model of the tumor microenvironment^9,10^.

Specifically, co-culture spheroids composed of cancer cells and fibroblasts serve as a useful model for studying tumor-stroma interactions^11^. While normal fibroblasts may inhibit tumor growth, for instance by secreting pro-apoptotic factors^12^, cancer-associated fibroblasts (CAFs) have been shown to support tumor progression and malignancy^13^. CAFs, activated by the tumor microenvironment (TME), mimic a chronic wound response, shifting from a protective to a tumor-promoting role^13,14^. Interestingly, the mechanisms underlying fibroblast motility within tumor environments remain poorly understood. In vitro co-culture spheroids may provide a valuable platform for investigating these fibroblast-tumor dynamics and could aid in identifying therapeutic targets^15,16^.

High-throughput imaging enables the acquisition and analysis of hundreds of biological samples within a single experiment, typically using automated imaging of multi-well plate formats such as 96-well plates. It supports monitoring over days under incubated conditions, at temporal resolutions of single seconds^17,18^. When applied to tumor spheroids, this enables simultaneous examination of multiple hypotheses in parallel, generating sufficient data for robust statistical analysis^19,20^. However, currently used high-throughput microscopes for spheroid imaging primarily produce images that contain only 2D information^1,17^. To fully characterize these 3D samples, multiple images along the z-axis need to be captured per sample, making the process time-consuming and limiting the practicality, throughput, and temporal resolution of large-scale experiments^21^. In addition, capturing numerous images per session increases the risk of phototoxicity and photobleaching due to extended light exposure^22^, and generates large data volumes that complicate storage and analysis. While confocal and light-sheet high-throughput systems can provide improved optical sectioning and resolution, they typically involve increased system complexity and cost^17,23^. Consequently, wide-field fluorescence high-throughput imaging remains a simple and affordable approach, motivating the development of methods that extend its capabilities to efficient 3D imaging with compact data acquisition.

The incorporation of point-spread function (PSF) engineering into such systems via compact optical modifications enables enhanced 3D imaging capabilities that have the potential to overcome these limitations using a minimal number of image acquisitions^24^. PSF engineering refers to the deliberate reshaping of the system point-spread function, namely the image formed by a point source, to encode additional information. Specifically, integrating a Tetrapod phase mask enables depth encoding within single images, facilitating compact data acquisition for 3D analysis^24–26^. However, algorithms for 3D localization or reconstruction in PSF-engineered systems have been developed primarily for point sources^27,28^ or domain-specific sparse objects^29–31^, rely on specialized cameras^32^, or require large-scale labeled datasets^24^.

Here, we reveal unique, previously unreported, spatially symmetric interaction patterns of green fluorescent protein (GFP)-expressing fibroblasts co-cultured with tumor spheroids, using high-throughput imaging across hundreds of conditions and time points. These patterns feature fibroblasts self-organizing into discrete clusters that distribute symmetrically around the spheroid surface in “flower-like” arrangements. To characterize these interactions in 3D using a minimal number of image acquisitions, we utilize a PSF-engineered high-throughput microscope and develop methods for snapshot or dual-shot 3D localization of extended fluorescent objects. The introduced methods rely solely on a PSF model, rather than training data. Our 3D analysis reveals that fibroblasts exhibit climbing and penetration into the spheroid, and identifies a characteristic 3D distance between adjacent fibroblast clusters. Furthermore, our approach facilitates rapid quantification and classification of drug responses that modulate these interaction patterns.

## Results

### Revealing a unique spatial organization in spheroid-fibroblast interactions

**Fig. 1:**
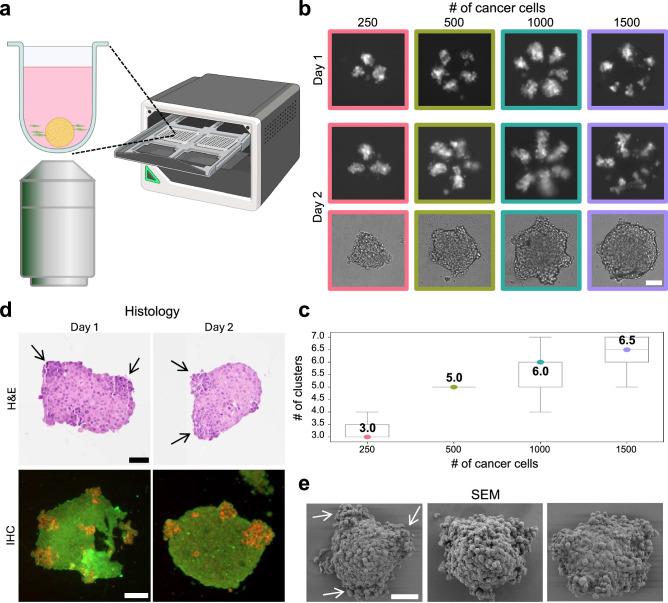
2D characterization of spheroid-fibroblast interaction patterns. **a** An illustration of the experiment setup - Incucyte® S3 is used for high-throughput imaging of spheroid interactions with fibroblasts. The zoom-in shows a well containing a single cancer cell spheroid (yellow), with GFP-expressing fibroblasts (green) approaching it, in liquid medium (pink). The illustration is schematic and not drawn to scale. The microscope and well images were created in BioRender. Bio, N. (2026) https://BioRender.com/sfqcjze. **b** Images of spheroid-fibroblast interactions focused on the spheroid center. Column labels indicate the number of seeded FaDu cells. The first and second rows show fluorescence images from day 1 (24 h) and day 2 (48 h), respectively; the third row shows bright-field images from day 2. For the representative images shown, the ratio of seeded fibroblasts to FaDu cells was 1:3 for 250, 500, and 1000 FaDu cells, and 1:10 for 1500 FaDu cells. Scale bar: 100 µm. **c** Box plot showing median values, illustrating the number of formed clusters vs the number of FaDu cells, demonstrating that more clusters are formed as spheroid size increases. The number of examined samples is *n* = 15, 14, 13, and 14 for 250, 500, 1000, and 1500 cancer cells, respectively. **d** Histology images of spheroid-fibroblast interactions imaged on days 1 and 2 with hematoxylin and eosin stain (H&E) and fluorescence immunohistochemistry (IHC), where fibroblasts appear in orange. Scale bars: 50 µm. **e** Scanning electron microscopy (SEM) images of spheroid-fibroblast interactions, imaged on day 1. Scale bar: 50 µm. In (**d**) and (**e**), arrows indicate suspected fibroblast clusters.

Using an Incucyte® S3 Live-Cell Analysis System (Sartorius), providing wide-field fluorescence and bright-field imaging in up to six 96-well plates, we imaged dozens of co-culture samples aiming to study the interactions between cancer spheroids and fibroblasts (Fig. 1a). We observed a unique, spontaneously organized, symmetrical interaction pattern between cancer spheroids and fibroblast clusters (Fig. 1b–e). In these experiments we used head and neck cancer cells (FaDu) or liver cancer cells (SK-136) to form spheroids in round bottom, ultra-low attachment (ULA) 96 well plates that promote the formation of a single, spherical spheroid per well, consistently positioned at the well center^33^. We then add fibroblast cells (3T3) to each well and examine their interaction. The Incucyte is placed in an incubator, enabling imaging over prolonged periods of time (hours to days) in physiologically relevant conditions (see “Methods”).

We examined the interaction patterns using 2D fluorescence microscopy over 48 h across different FaDu spheroid sizes (250, 500, 1000, and 1500 seeded FaDu cells per well) and varying numbers of fibroblast cells to maintain 3T3:FaDu ratios of 1:3, 1:5, and 1:10 (Supplementary Note 1). The interactions resulted in symmetrical flower-like shapes (Fig. 1b) that are notably robust, reproducible, and consistent (see Supplementary Note 1 for all 96 samples imaged on a single plate).

We initially discovered that the major factor affecting the number of formed clusters (obtained using cluster segmentation; Supplementary Note 1) is the spheroid size, or equivalently, the number of seeded FaDu cells, with more clusters forming as spheroid size increases (Fig. 1c). Interestingly, the ratio of added fibroblast did not affect the number of formed clusters, but only their size. This trend was observed consistently across most tested conditions, with only minor deviations at certain fibroblast-to-spheroid ratios (Supplementary Note 1).

These patterns were further characterized using histological staining and scanning electron microscopy (SEM), performed 24 or 48 h after the addition of fibroblasts (Fig. 1d, e). The images show fibroblast clusters integrating with the spheroid structures, validating the interaction patterns revealed in our fluorescence microscopy images.

### PSF-engineered high-throughput microscopy for 3D cell localization

**Fig. 2:**
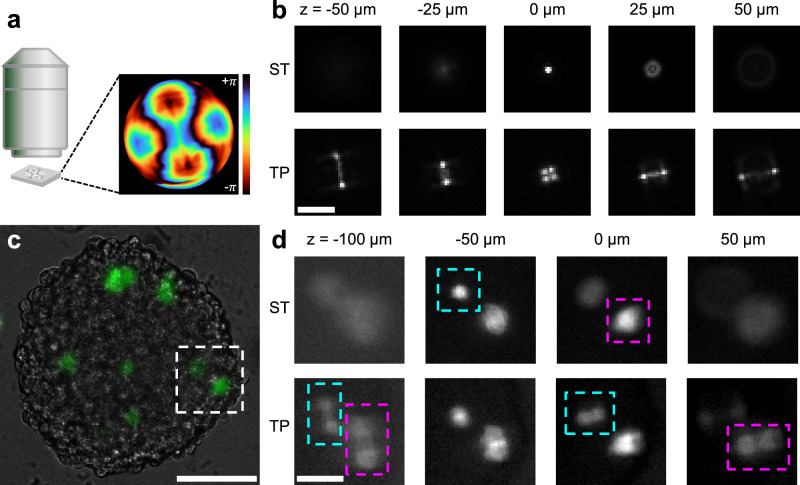
PSF engineered system for imaging cell-spheroid interactions. **a** Left: Objective lens with a Tetrapod (TP) phase mask at its bottom^24^. Right: The TP phase mask profile. **b** Z-stacks of a fluorescent bead using both standard (ST) and TP objectives. *z* = 0 µm is the bead focus plane. Scale bar: 20 µm. **c** An image of a non-stained SK-136 cancer spheroid interacting with GFP-expressing fibroblasts. The image combines ST bright-field and green-channel images focused on the axial center of the spheroid (see “Methods”). Scale bar: 100 µm. **d** Zoom-in on the patch marked in (**c**), showing green-channel images at different objective positions (*z*). The reference plane, defined as *z* = 0 µm, corresponds to the axial center of the spheroid. A single cell and a cell cluster are marked in cyan and magenta dashed boxes, respectively, highlighting the TP effect in defocus compared to the ST focused image. Scale bar: 20 µm.

Next, we aimed to characterize the formation of the interaction patterns and the distances between adjacent clusters in 3D. While z-stack acquisition could potentially provide such information, in time-lapse imaging over hours or days, this extensive acquisition would expose the sample to prolonged illumination, causing phototoxicity and photobleaching (Supplementary Note 2), thereby compromising sample viability and physiological relevance. In addition, it would degrade temporal resolution since it limits the number of samples we can examine in a certain time frame, and it would increase the amount of collected data. Therefore, to obtain high-throughput 3D imaging with a minimal number of images, we encode depth in the captured images by utilizing PSF engineering. This is done by the incorporation of a Tetrapod (TP) phase mask at the bottom of the objective^24^ (Fig. 2a; see “Methods”).

The effect of the Tetrapod phase mask on the 3D PSF of the system is shown in Fig. 2b, displaying a z-stack of a fluorescent bead. The axial position of the bead is encoded in the strongly z-dependent shape of the PSF, where the informative depth of field (DOF) is substantially extended compared to a standard (ST) objective. The increase in DOF and improved axial resolution come at the expense of some lateral resolution degradation, because the object is convolved with a PSF with lateral size of ~10–20 µm (Fig. 2b). For our purposes of determining the positions of cells and clusters, this resolution is sufficient. In our experimental setup, the DOF expands to 225 µm z-range in the sample (see Supplementary Note 3 for a discussion considering refractive index mismatch). The spheroid diameters range from 200 µm to 300 µm, so that the informative DOF covers a range larger than the imaged bottom half of the spheroid.

Imaging fluorescent cells, which are extended, non-point objects, using the Tetrapod phase mask also contains 3D information (Fig. 2c, d). A single cell generates a shape that resembles the TP pattern, albeit blurry due to the size of the cell. The images of larger cell clusters are composed of more arbitrary structures, in which case the 3D information appears more implicitly, e.g., by lateral stretching of the cluster.

The differences between the two cases, i.e., single-cells and cell-clusters, require different imaging acquisition and reconstruction procedures. Specifically, motivated by our desire to achieve 3D imaging using a minimal number of images, we developed single-shot and dual-shot methods for 3D localizations of single cells and cell clusters, respectively.

### Single-shot 3D cell localization using maximum-likelihood estimation reveals fibroblast-spheroid interaction trajectories

To study the interaction between spheroids and single fibroblasts in 3D over time, we initially used a low concentration of fibroblasts (less than ten fibroblasts per spheroid), appearing as single cells throughout the process. To determine the 3D position of an individual fibroblast with respect to the spheroid, we developed a maximum-likelihood estimation (MLE) based method requiring only a single image, with no z-scanning (Supplementary Notes 3 and 4).

**Fig. 3:**
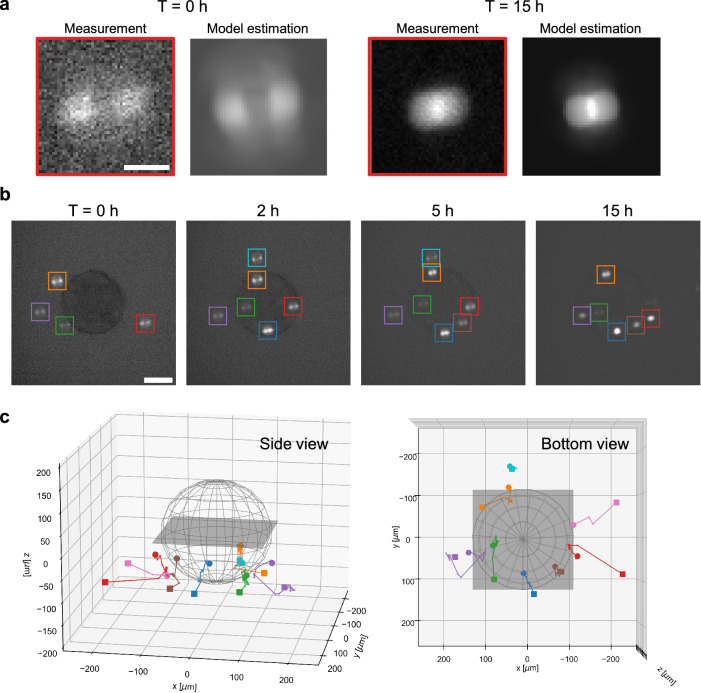
3D tracking of cell-spheroid interactions using single-shot MLE-based 3D cell localization. **a** Cell TP measured images and their corresponding model estimations at different imaging times *T* = 0, 15 h. The *z* values estimated by the model are −106 µm and −49 µm for *T* = 0 h and *T* = 15 h, respectively. The images correspond to the cell marked in red in (**b**). Scale bar: 20 µm. **b** Time-lapse imaging of spheroid-cell (FaDu-3T3) interactions using the TP objective. All images were captured at a consistent focus depth. The spheroid outline is visible. The boxes highlight cells undergoing TP shape changes over time, indicating changes in their depth. Each color represents a different cell. Scale bar: 100 µm. **c** 3D reconstruction of the interactions, obtained using the MLE algorithm from a single image acquired at each time point. Each color represents the corresponding cell in (**b**), where squares and circles mark the initial and final tracking points, respectively.

The MLE-based cell localization is based on a numerical model that generates a cell TP image, given the 3D coordinates of the cell, the cell intensity, and the background noise level. This forward cell-imaging model is used to solve the inverse problem of obtaining the cell image parameters mentioned above, including z-depth (Fig. 3a), by MLE. The imaging model relies on VIPR, a phase retrieval method^28^, to obtain the PSF model from bead measurements, which is then convolved with a circle to generate the defocused cell images (see “Methods”). The model assumes a single circular shape for all standard in-focus images of single cells, which is a reasonable assumption because single-cell images are sufficiently similar. However, this is not suitable for cell clusters, which exhibit arbitrary shapes depending on the number of cells they contain and their spatial arrangement (Fig. 2d). To address this limitation, we developed a dual-shot method for cell cluster z-localization (see next section).

The developed method enables 3D localization of single cells with an accuracy of 9 µm, which is smaller than the size of a single cell (Supplementary Note 5)^34^. Importantly, this localization error is small relative to the size of the spheroids (200–300 µm in diameter), enabling accurate characterization of individual cell trajectories as they approach and interact with the spheroids. We further compared our method to localizations using ST imaging. Using the ST PSF, only ~40% of the cells are fittable, due to the limited depth encoding and smaller depth of field of the ST PSF. Additionally, using ST z-stacks requires dense axial sampling with Δz < 45 µm (in the sample) to reach similar accuracy. This results in the acquisition of at least 3–6 images to cover a z-range of 90–225 µm in the sample (see Supplementary Note 6 for both results). Overall, our ability to perform 3D cell tracking using a single shot per time point with the TP PSF enabled tracking of dozens of samples in a single experiment with enhanced temporal resolution and lower phototoxicity compared to z-stack acquisitions.

Figure 3b shows a sample in which a few fibroblasts interact with a spheroid (see also Supplementary Video 1). The 3D reconstruction presented in Fig. 3c, based on single snapshots, illustrates the cell trajectories as they approach and interact with the spheroid, with localizations linked via linear interpolation. Surprisingly, there is a clear trend of cells starting from the bottom of the well and, after attachment, climbing along the spheroid until they reach a certain height on the spheroid surface (Fig. 3c). At high fibroblast concentrations, this forms the basis towards fibroblast clusters forming on the spheroid surface, at a highly regular spacing, and their invasion over time, which we show later. This is, to the best of our knowledge, the first report of this phenomenon.

### Dual-shot depth-from-defocus based method for 3D cell cluster localization

We next examined fibroblast-spheroid interactions using a higher concentration of fibroblasts, resulting in the formation of fibroblast clusters. For cell clusters, single-shot MLE z-localization is not suitable because of the wide variability in cell cluster shapes, which depends on the cluster size and structure (Fig. 2d). Therefore, to determine the 3D positions of clusters, we developed a method that utilizes two images. The additional information provided by two images, rather than one (as in the MLE method), alleviates the need for explicit cluster-shape estimation. Our dual-shot method is based on the depth-from-defocus (DfD) concept, in which depth is obtained by utilizing defocus information^35,36^, which in our case involves optical encoding by the TP PSF.

**Fig. 4:**
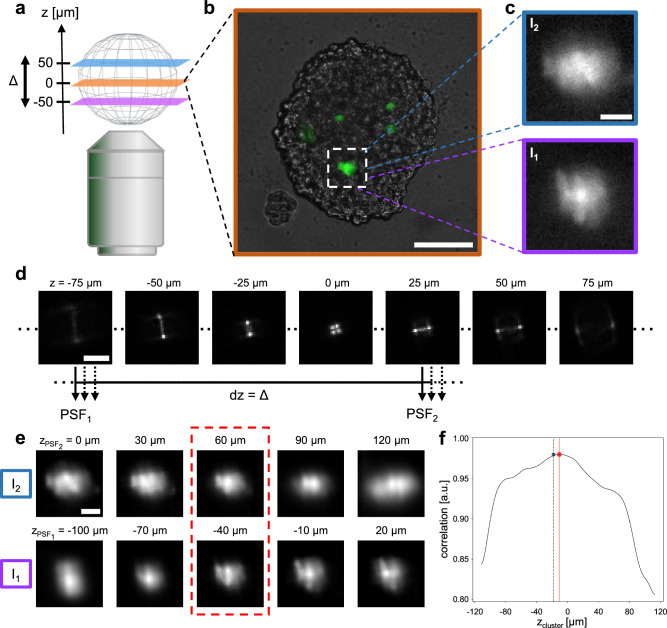
Dual-shot DfD method for cell cluster 3D localization. **a** Illustration of imaged z-slices, specified by objective *z*-positions. Orange slice – spheroid axial center, *z* = 0 µm; Purple and blue slices – bottom (*z* = −50 µm) and top (*z* = 50 µm) slices, respectively, with Δ representing the *z*-distance between them. **b** SK-136 spheroid image at *z* = 0 µm, combining an ST bright-field image and a TP green-channel image, with the examined 3T3 cell cluster highlighted by a white dashed box. Scale bar: 100 µm. **c** Images of the examined cell cluster at *z* = −50, 50 µm, acquired with the TP objective, labeled as *I*_1_ and *I*_2_, respectively. Scale bar: 20 µm. **d** Measured bead PSFs. Each pair of PSF_1_ and PSF_2_ is used to deconvolve *I*_1_ and *I*_2_, respectively. The z-distance between PSF_1_ and PSF_2_ is Δ. Scale bar: 20 µm. **e** Deconvolution results for *I*_1_ and *I*_2_ using PSF_1_ and PSF_2_, respectively, for distinct PSF *z*-values. For each pair of deconvolved images of *I*_1_ and *I*_2_ the *z*-distance between z_PSF,1_ and z_PSF,2_ is Δ. The red dashed box marks the pair of deconvolved images of *I*_1_ and *I*_2_ that are most similar. Scale bar: 20 µm. **f** Correlation between the deconvolved images of *I*_1_ and *I*_2_ across z_cluster_ values; the peak correlation yields an estimated z_cluster_ of −10.5 µm (red), while the ground-truth estimation is −18 µm (black).

We use two TP images acquired below and above the spheroid axial center, from which clusters are cropped and localized (Fig. 4a–c and Supplementary Note 4). The depth of a single cluster, relative to the imaging planes, is encoded in these two PSF-engineered images. To extract the depth information, we use a PSF model, based on bead measured PSFs (Fig. 4d), and seek the PSF pair that best corresponds to the measured images. Finding the depth of the cluster that is most consistent with the PSF-encoded image pair relies on Lucy-Richardson deconvolution^37,38^ using the PSF model (Fig. 4e, f; see “Methods”). This dual-shot method achieves an estimated accuracy of 12 µm (Supplementary Note 7), which is comparable to the size of a single cell^34^, enabling accurate 3D characterization of fibroblast cluster organization and interactions with spheroids. For comparison, localizations using ST imaging require z-stack acquisitions with Δz < 45 µm (in the sample), increasing the number of required images to at least 6 for the examined z-range (similarly to the MLE method comparison to localization using ST imaging; see Supplementary Note 6).

### Characterizing spheroid-fibroblast interactions in 3D

**Fig. 5:**
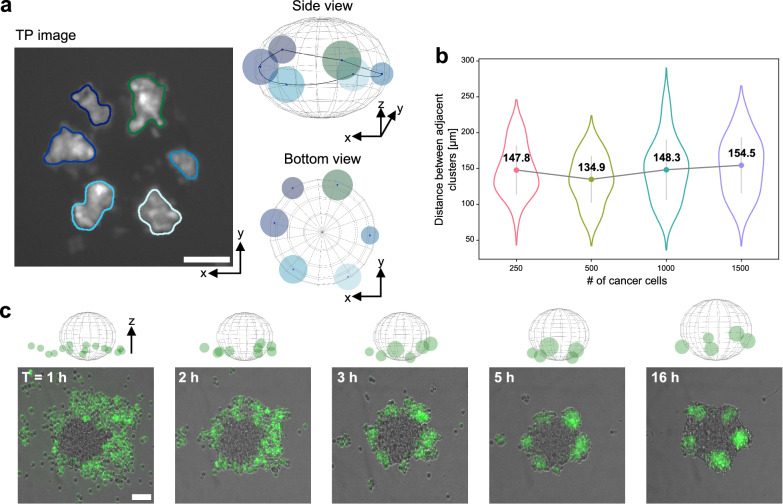
3D characterization of spheroid-fibroblast interaction patterns. **a** Illustration of spherical distances between adjacent clusters. 3D reconstructions of the spheroid represented in the Tetrapod (TP) image are shown in side and bottom views. Each color represents a different cluster. Arcs connecting clusters indicate the calculated spherical distances along the spheroid surface. Scale bar: 100 µm. **b** Violin plot showing 3D spherical distances between adjacent clusters for different numbers of FaDu cells, indicating an approximately constant characteristic distance of 146 µm. The number of adjacent cluster pairs is *n* = 32, 42, 44, and 31 for 250, 500, 1000, and 1500 cancer cells, respectively. **c** Generation of the flower-like interaction pattern over time. Bottom row: Composite images combining a TP bright-field image and a TP green-channel image focused on the spheroid axial center captured at various time points: 1, 2, 3, 5, and 16 h. Top row: Corresponding 3D reconstructions. Scale bar: 100 µm.

Using our DfD method for cell cluster localization, we characterized the 3D structure of the flower-like pattern we introduced previously. For sample characterization, fibroblast clusters were segmented, and their 2D weighted centers and total areas were obtained. Next, clusters were cropped for DfD analysis, yielding their *z* locations. Using the obtained 3D localizations we then calculated the 3D spherical distances, namely, the distance on a sphere, along the great-circle arc, between adjacent clusters (Supplementary Note 8). This analysis revealed a characteristic distance of approximately 146 µm, independent of the amount of seeded FaDu cells or ratio of added fibroblasts (Fig. 5a, b and Supplementary Note 8). Although a defined spacing between fibroblast clusters interacting with spheroids has not been mentioned in previous research, fibroblasts in 3D matrices or 3D cell cultures have been shown to influence each other over long distances through mechanical coupling^39^, ECM reshaping^40^, and contact-inhibition^41^. Moreover, their dynamic behavior was introduced in a “fibroblast-led invasion” model, where fibroblasts guide coordinated cancer cell migration from spheroids to ECM environment^42^.

We hypothesized that the patterns formed from interactions between fibroblast clusters and spheroids are primarily driven by the fibroblasts, rather than by the geometry of the spheroids. To test this, we examined the interaction of fibroblasts with large, irregularly shaped spheroids (approximately 1 mm in length). We detected, in this case as well, the formation of distinct fibroblast clusters, indicating that their formation is not caused by the symmetric spheroid shape (Supplementary Note 9). To further examine our hypothesis, we added a different head and neck cancer cell line, Cal33, instead of fibroblasts, to FaDu spheroids. Under these co-culture conditions, clusters were not generated. Instead, Cal33 cells spread in a relatively uniform pattern around the spheroid (Supplementary Note 10). Both results support our hypothesis, indicating that the formation of the pattern is mainly driven by the fibroblasts. The tendency of fibroblasts to attach and penetrate as clusters into tumor spheroids, rather than forming isolated clusters or a layer around the tumor cells, is likely mediated by chemotactic signals, integrin-ECM binding, and tumor-derived paracrine cues that mimic a wound-healing environment^43,44^.

To test whether the flower-like pattern also occurs with other cancer types, we examined SK-136 spheroids. Fibroblasts formed clusters, but with reduced symmetry and deeper penetration compared to FaDu spheroids. This behavior is not very well understood and is likely due to SK-136’s different chemokine secretion, structural integrity, and density (Supplementary Note 11).

To observe the formation of the flower-like pattern over time, we tracked and reconstructed the process in 3D (Fig. 5c and Supplementary Note 8). Tracking was performed using our DfD method by dual-shot acquisition per time point. Our results indicate that the clusters are formed and arranged at an early stage of the interaction (*T* = 3 h), around the time the fibroblasts begin to gather around the spheroids. Once initiated, clusters move to ultimately form a symmetrical structure (*T* = 5 h), where fibroblasts tend to join the nearest cluster (another example is shown in Supplementary Video 2). Clusters then rise towards the spheroid axial center, ultimately penetrating towards its core (*T* = 16 h). Importantly, we noticed that the majority of fibroblasts are imaged and not occluded by the spheroid, as they tend to initially gather around the bottom half of the spheroid (Supplementary Note 12).

### The effect of drugs on fibroblast-spheroid interaction patterns

We further examined the effects of different drug treatments over time on spheroid-fibroblast interactions. Our goal was to identify drugs that modulate these interactions, both to provide biological insights and to explore potential therapeutic applications. Specifically, a drug that reduces the mobility of fibroblast cells could serve as a basis for developing treatments for inhibiting fibrosis^45^ or for inhibiting tumor progression by disrupting fibroblast-assisted tumor growth^46,47^. Conversely, our method could also be used to identify drugs that enhance cell infiltration dynamics, enabling modified cells designed to penetrate solid tumors to infiltrate more effectively. These include immune cells like macrophages and T-cells^48,49^ or genetically engineered cells carrying drugs^50,51^. Tracking was performed as in the flower generation process (Fig. 5c), enabling 3D characterization using dual-shot acquisition per time point.

We examined six drugs that have been previously reported in the literature with relation to cell mobility or that have been previously published as inhibiting cell invasion to tumors. These drugs were initially evaluated in a two-dimensional wound healing assay with fibroblasts, providing an efficient platform for preliminary assessment of their effect on cell motility (Supplementary Note 13). Then, the selected drugs were added to the spheroid-fibroblast co-culture samples, which were seeded at a consistent 1:10 ratio of 3T3 to FaDu cells (100:1000 per well) and were imaged using our system (see Supplementary Note 14 for all 96 samples imaged on a single plate in this experiment). Ultimately, we focused on the drug ponatinib, an FDA-approved multi-kinase inhibitor for chronic myeloid leukemia^52^, with some reports indicating its activity on cell motility and invasion^53–55^. This choice was based on its significant effect on fibroblast interactions with FaDu spheroids (Supplementary Notes 14 and 15).

**Fig. 6:**
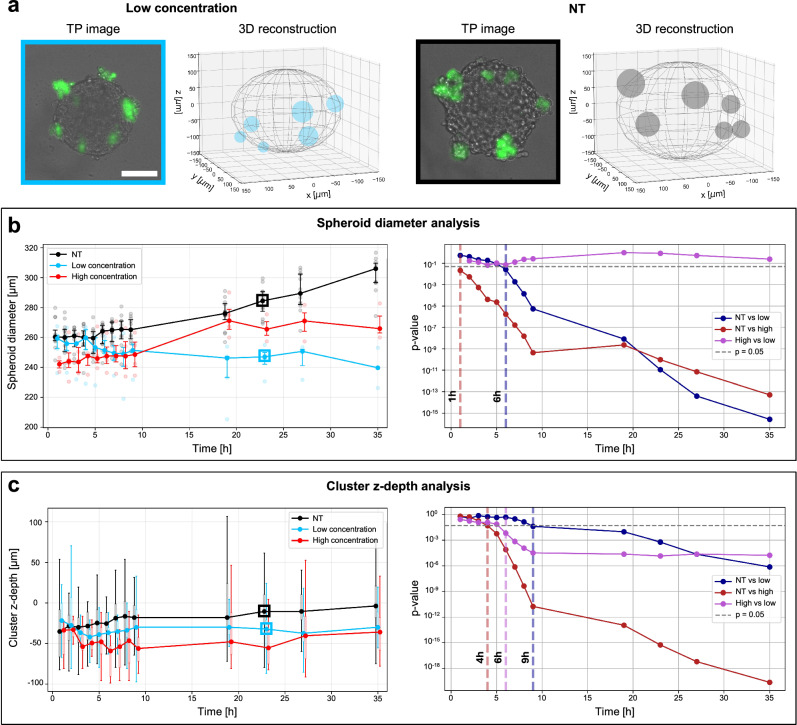
Drug effect on spheroid-fibroblast interactions. **a** Tetrapod (TP) images and 3D reconstructions of spheroid-fibroblast interactions under low concentration of the drug ponatinib (5 · 10^−4^ mg/ml) vs untreated (NT) samples, at *T* = 23 h. TP images combine an ST bright-field image and a TP green-channel image focused on the spheroid center. Scale bar: 100 µm. **b** Spheroid diameter analysis. Left: Spheroid diameters over time, for different drug concentrations - NT, low (5 · 10^−4^ mg/ml), and high (5 · 10^−3^ mg/ml). Squares indicate the populations from which the samples shown in (**a**) were extracted. At each time point, dark points represent median values, error bars indicate interquartile range (IQR; shown only for *n* ≥ 3), and light points represent individual measurements. Right: Statistical analysis showing *p*-values as a function of time, calculated using Hotelling’s *T*^2^ test for the joint distribution of spheroid diameter and time for each drug concentration pair. The first time point at which the *p*-value drops below 0.05 is marked: 1 h for NT vs high (*p*-value = 0.021) and 6 h for NT vs low (*p*-value = 0.026). For high vs low, the *p*-value did not drop below 0.05. **c** Cluster z-depth analysis. Left: Cluster z-depth over time for the same drug concentrations shown in the left plot of (**b**) - NT, low and high. Squares indicate the populations from which the samples shown in (**a**) were extracted. At each time point, dark points represent median values, and box plots show the distribution. Right: Statistical analysis showing *p*-values as a function of time, calculated using Hotelling’s *T*^2^ test for the joint distribution of cluster z-depth and time for each drug concentration pair. The first time point at which the *p*-value drops below 0.05 is marked for each comparison: 4 h (NT vs high; *p*-value = 0.048), 6 h (high vs low; *p*-value = 0.006), and 9 h (NT vs low; *p*-value = 0.04). In the analyses shown in (**b**, **c**), 12, 4, and 5 samples were examined for NT, low, and high drug concentrations, respectively.

To test the effect of ponatinib on spheroid-fibroblast interaction patterns, we characterized the samples under two drug concentrations, low (5 · 10^−4^ mg/ml) and high (5 · 10^−3^ mg/ml), and compared them to untreated (NT) samples over time (Fig. 6a and Supplementary Note 14). First, we found that the spheroid diameter measured during the first 6 h was inversely correlated with drug concentration. At the high concentration, we observed apparent spheroid size expansion over time, driven by toxic effects that altered morphology and led to disassembly, rather than proliferative expansion. In contrast, at the low concentration, spheroid growth was inhibited compared to untreated controls (Supplementary Note 15). In addition to sample characterization, we tested whether spheroid diameter could be useful for classification of drug concentrations. To do so, we performed a multivariate Hotelling’s *T*^2^ test between each pair of drug concentration conditions (NT vs low, NT vs high, high vs low). We calculated *p*-values for the joint distribution of two variables, spheroid diameter and time, and found that differences between NT vs high concentration and NT vs low concentration were statistically significant (*p* < 0.05) after 1 and 6 h, respectively (Fig. 6b). This suggests that spheroid diameter could be a useful parameter for classification of drug concentrations in these cases.

Spheroid diameter measurements alone could not statistically distinguish between high and low drug concentrations (Fig. 6b). However, using the capability of our system to provide 3D information, we made an interesting observation: fibroblast clusters, as analyzed using our DfD algorithm, tended to be located at lower z-positions under higher ponatinib concentrations.

This finding is consistent with the mechanism of ponatinib, a multi-targeted tyrosine kinase inhibitor that potently inhibits SRC family kinases, FGFR, PDGFR, and VEGFR^52,56^. Fibroblast climbing on spheroid surfaces requires dynamic focal adhesion turnover and integrin-mediated adhesion to navigate the spheroid’s curved geometries. SRC kinases play a crucial role in integrin signaling and regulate cell migration by phosphorylating focal adhesion proteins^57^, while PDGFR signaling coordinates cell proliferation, survival, adhesion, and migration^58^, all critical processes for fibroblasts to establish traction and ascend the spheroid surface. By inhibiting these pathways, particularly SRC and PDGFR, ponatinib reduces cell migration and inhibits normal cell motility functions^52,56^, preventing fibroblasts from initiating their climb before they can establish mature attachments needed for penetration.

Importantly, cluster z-depth enabled early distinction between high and low drug concentrations, which could not be achieved using spheroid diameter alone. This result was obtained using a similar multivariate Hotelling’s *T*^2^ test, including cluster z-depth instead of spheroid diameter (Fig. 6c). This analysis highlights the potential added value of the cluster z-depth parameter for fast, sensitive classification of novel drug effects, such as inhibiting the climbing from the bottom of a sphere.

## Discussion

In this work, we uncover unique “flower-like” interaction patterns of fibroblasts with tumor spheroids with surprising trajectories across dozens of samples in each experiment using high-throughput 3D analysis. The observed interaction pattern features a characteristic distance between adjacent fibroblast clusters, which appears independent of spheroid size and fibroblast number, provided a sufficiently large number of fibroblasts are present. The methods we introduce for single-shot and dual-shot 3D localization enable volumetric characterization with enhanced temporal resolution and imaging throughput, reduced phototoxicity and photobleaching, and lower data storage, compared to z-stack acquisition. Additionally, our methods are unsupervised and rely solely on a PSF model, eliminating the need for training data or labeled examples. These capabilities make the methods particularly well-suited for preclinical drug screening in spheroid-based samples and for studying interactions between spheroids and surrounding cell populations in dynamic 3D environments.

We observed that the fibroblast penetration profiles into cancer spheroids depend mostly on the cancer cell line, however, in all cases, the fibroblasts are drawn to the spheroid center. We hypothesize that this behavior is partially driven by the necrotic nature of the spheroid core and that the characteristic 146 μm spacing between fibroblast clusters suggests an active self-organization mechanism rather than random distribution. Given their physiological role in wound healing^59^, fibroblasts likely interpret the necrotic spheroid core as a chronic wound-like environment^60^, triggering coordinated migration patterns.

The consistent inter-cluster spacing observed in our system may reflect coordinated fibroblast behavior mediated by biomechanical and adhesive interactions, rather than a purely stochastic distribution^61^. One plausible explanation is mechanical coupling through ECM remodeling, whereby fibroblast-generated traction forces locally reorganize the matrix and influence neighboring clusters within a finite interaction radius^62,63^. Actomyosin-dependent tension, calcium-dependent adhesion^64^, and dynamic protrusive activity (filopodia or lamellipodia^65^) may contribute to this coordinated organization. In addition, paracrine signaling^66^, contact inhibition of locomotion^67^, and matrix-dependent feedback^68^ could participate in shaping the spacing pattern.

The precise mechanism underlying this spatial periodicity remains to be elucidated, and further mechanistic studies will be required to establish causality. A deeper understanding of these processes may ultimately inform strategies to modulate fibroblast infiltration in pathological contexts such as tumor progression and fibrosis^69,70^.

Our method also provides insights into the 3D localization of fibroblast clusters interacting with spheroids under drug influence. Incorporating the z-depth parameter into the statistical analysis enables early detection of drug effects, highlighting the added value of 3D information over conventional 2D imaging for this task. Specifically, our z-depth analysis revealed a previously uncharacterized phenomenon: ponatinib inhibits the initial “climbing” phase of fibroblast-spheroid interaction, where fibroblasts ascend the curved spheroid surface before penetrating inward. This likely reflects broad inhibition of kinases by ponatinib, particularly SRC and PDGFR pathways, which are essential for cell motility. Our 3D depth tracking captures this early spatial effect within the first 2–4 h, substantially earlier than traditional viability assays or spheroid diameter measurements detecting drug influence, demonstrating the enhanced temporal sensitivity of volumetric analysis for characterizing drug effects on complex cell-cell interactions.

Similar to other microscopic imaging techniques, we encounter challenges when imaging cells located within spheroids, which are composed of densely packed cellular structures. This dense packing increases light scattering, limiting our ability to clearly image fibroblast clusters that are integrated into the spheroid structure. As a result, the cluster images appear blurred, which reduces localization accuracy. This issue becomes more pronounced in larger spheroids, where increased tissue thickness further scatters the light before it reaches the objective, leading to a further decline in imaging resolution. In addition, fibroblasts located above the spheroid may be occluded, making their fluorescence challenging to detect, with the limitation becoming greater as spheroid size increases. As we show, this effect is minimal in our samples.

The methods we present for 3D localization consist of single-shot imaging for single cells and dual-shot imaging for cell clusters. Single-shot acquisition provides high temporal resolution while minimizing phototoxicity and reducing data volume, compared with 3D imaging based on z-stack acquisition. For cell clusters, the use of dual-shot imaging reduces the achievable temporal resolution relative to single-shot imaging and requires twice the illumination exposure and data acquisition. Nevertheless, dual-shot acquisition still offers substantial advantages over z-stack acquisition. In our experiments, the observed processes evolve on timescales of minutes to hours, which are well captured by two sequential acquisitions.

Moreover, the accuracy of our 3D localization methods is influenced by the cluster size. Our approach estimates a single mean axial position for each volumetric cluster based on its 3D-encoding images, which contain overlapping signals from all the cells within the cluster. As cluster size increases, the internal variation in cell positions also grows, and the resulting mean depth estimate tends to be biased toward the brightest regions of the cluster rather than its geometric center, or toward cells located closer to the objective, as scattering reduces the contribution of deeper cells to the overall image. This bias becomes more pronounced with increasing cluster size, compromising localization fidelity.

In addition, the TP PSF extension of the DOF and improvement of the axial resolution comes at the expense of reduced lateral resolution. The degradation becomes more severe for clusters than for single cells, and becomes worse for larger clusters, because signals from individual cells within a cluster mix and overlap, making them more challenging to resolve.

Throughout this work, we used a Tetrapod phase mask designed to encode an axial range of 150 µm in a single image. An open question remains as to whether this phase mask configuration is optimal for tracking 3D interactions between submillimeter spheroids and surrounding cells. Future research could explore alternative Tetrapod designs^26^, such as extending the axial range to cover larger spheroids, or increasing the lateral separation between the PSF lobes to reduce image overlap, for more distinguishable cluster duplicates. A complementary direction involves a more task-specific design approach, in which neural networks can be used to learn an optimal phase mask tailored to a particular imaging application. This framework can also incorporate a post-processing stage that decodes the resulting images to recover relevant information encoded by the learned mask. The phase mask design and post-processing step can also be jointly optimized in an end-to-end manner^27,71–73^. These approaches require explicitly defining the task objective during optimization. Examples of such objectives, beyond 3D localization, include counting individual cells within clusters, generating extended depth-of-field projection images of multicellular structures, and simultaneously resolving 3D positions while producing focused images of clusters.

Beyond the current study, our work could be naturally extended to enable 3D analysis of interactions between spheroids and other cell types in the TME, such as immune cells (CAR T-cells, for example) and endothelial cells. Moreover, the 3D cluster localization methods we present are broadly applicable and, beyond spheroids, could be adapted for a wide range of biological samples, including organoids, microtissues, and 3D printed models, imaged using high-throughput microscopy.

## Materials and methods

### Materials and reagents

All non-drug chemicals were purchased from Sigma Aldrich (St. Louis, MO, USA). DMSO was purchased from Carlo Erba (Emmendingen, Germany). Drugs were purchased from LC-Laboratories (Woburn, MA, USA) and Tzamal Medical Group (Petach Tikva, Israel).

### Cell cultures

The SK-136 cell line originated from murine hepatoblastoma cells with amplified c-Myc and β-catenin, obtained and cultivated in the lab of Daniel Heller, Memorial Sloan Kettering Cancer Center, USA, which kindly shared them with us^74^. The 3T3-ZsGreen expressing fibroblast cell line originated from mouse embryonic fibroblasts, and was also received from Daniel Heller’s lab. The FaDu cell line originated from a human hypopharyngeal squamous cell carcinoma in a male. The Cal33 cell line originated from a human tongue squamous cell carcinoma in a male. FaDu and Cal33 cell lines were generously provided by the lab of Moshe Elkabets, Ben-Gurion University, Israel. The cell lines were not independently authenticated and were not tested for mycoplasma contamination.

All cell lines were cultured in Dulbecco’s Modified Eagle Medium (DMEM) supplemented with 10% Fetal Bovine Serum (FBS), 2 mM L-glutamine, Penicillin G Sodium Salt: 100 units/ml, and Streptomycin Sulfate: 0.1 mg/ml (all purchased from Biological Industries, Israel). All cells were incubated at 37 °C with 5% CO_2_ and 65% humidity.

### Cancer spheroid-fibroblast co-culture

For single cell tracking and cluster localization: approximately 1000 FaDu cells or 500 SK-136 cells were seeded per well, in 96 round bottom ultra-low attachment (ULA) plates, and were given 24 h to form an initial spheroid structure before adding approximately 10 3T3 cells to each well. For spheroid-fibroblast interaction pattern characterization: FaDu cells were seeded at densities of 250, 500, 1000, or 1500 cells per well and incubated for 24 h to allow spheroid formation. Subsequently, 3T3 cells were added at varying 3T3:FaDu ratios of 1:3, 1:5, and 1:10. For inhibiting fibroblast invasion using drugs: approximately 1000 FaDu cells were seeded per well in 96-ULA round bottom plates and were left overnight to form spheroids. Then, 100 3T3 cells were added to each well and were given 1 h to immerse before drugs were added at 6 concentrations ranging from 3.7 · 10^−7^ to 0.01 mg/ml. The spheroids were imaged (bright-field and green-channel TP) every one to several hours for 4 days.

### Sample characterization using SEM and histology

For high-resolution scanning electron microscope (HR-SEM) imaging: spheroids were placed on poly-l-lysine-coated plastic coverslips (Thermonex) and fixed in 1% paraformaldehyde (PFA) and 2% Glutaraldehyde in 0.1 M cacodylate buffer for 1 h, rinsed in cacodylate buffer, and then incubated in 1% OsO_4_ in cacodylate buffer for 15 min at room temperature. Dehydration was carried out through a graded ethanol series: 8%, 15%, 30%, 50%, 70%, 90%, 95%, and through absolute alcohol. The samples were then dried using a Critical Point Dryer. The coverslips were mounted on SEM stubs and images were obtained with an Ultra-Plus high-resolution SEM (Zeiss) at the Faculty of Chemical Engineering, Technion, Israel. The samples were imaged at acceleration voltages of 0.7 or 0.8 kV and a working distance of 4.2 or 3.9 mm.

For histological staining: 1 · 10^6^ FaDu cells were added to ULA flasks and incubated for 24 h to allow spheroid formation. Subsequently, 3.3 · 10^5^ 3T3 cells were added to the flask for an additional 24 or 48 h to allow penetration into the spheroids. The spheroids were then fixed in 4% PFA overnight and washed 3 times with PBS, then embedded in paraffin. 3 μm slices were stained with hematoxylin and eosin stain (H&E). Images were taken using Olympus VS200 slide scanner at the Biomedical Core Facility (BCF), Technion, and analyzed with OlyVIA software.

In addition, antigen retrieval was performed by heating sections in citrate buffer (pH 6.0) using an 850 W domestic microwave at 80% power for 5 min, followed by incubation in a 95 °C water bath for 15 min. Permeabilization was carried out in 0.3% Triton X-100 in Tris-buffered saline (TBS; Sigma-Aldrich) for 10 min. Sections were blocked with 20% donkey serum (D9663, Sigma-Aldrich) in TBS for 30 min, then incubated overnight at 4 °C with primary antibodies against vimentin (ab24525, polyclonal antibody (Chk pAb), LOT 1091824-14, Zotal) and Nectin-4/PVRL4 (MABT64, clone N4.61, LOT 3744860, 1 mg/ml, Sigma-Aldrich) diluted 1:100 with donkey serum in TBS. Secondary antibodies (goat anti-chicken Alexa Fluor® 594, ab150176, Goat pAb to Chk IgY, LOT GR3401044-2, 2 mg/ml, Zotal; goat anti-mouse Alexa Fluor® 488, ab150117, Goat pAb to Ms IgG, LOT GR3398339-4, 2 mg/ml, Zotal), diluted 1:200 in TBS with 1% bovine serum albumin (BSA), were added for 1 h at room temperature. The anti-Nectin-4/PVRL4 antibody has been validated for the detection of human Nectin-4 and is used in immunohistochemical applications. Nectin-4 is highly expressed in many epithelial cancers, including head and neck squamous cell carcinomas, such as the FaDu cell line. Human Protein Atlas data indicate that the FaDu cell line expresses Nectin-4 at a relatively high level (13.8 normalized transcripts per million (nTPM); expression across cell lines reaches approximately 60 nTPM). Therefore, the antibody was expected to specifically label FaDu epithelial cancer cells of human origin, while showing no reactivity toward murine 3T3 fibroblasts. The anti-vimentin antibody has been validated by the manufacturer for immunocytochemistry/immunofluorescence (ICC/IF) applications in human, mouse, and rat samples. Vimentin is a well-established mesenchymal marker that is strongly expressed in fibroblasts, while FaDu cells exhibit low expression according to Human Protein Atlas data (2.7 nTPM), placing them among the lower-expressing cell lines in the dataset, where expression levels ranged up to approximately 14,000 nTPM. It was expected to strongly label 3T3 fibroblasts and potentially a subset of FaDu cells exhibiting mesenchymal characteristics. Slides were counterstained with Hoechst, mounted, and imaged using a LionHeart FX automated fluorescence microscope (BioTek, Agilent Technologies, Santa Clara, CA, USA).

### Bead samples

Bead samples were prepared using green fluorescent beads (G0100, Thermo Fisher Scientific) with a diameter of 1 µm, diluted to a concentration of 10^−4^ in 1% poly(vinyl alcohol) (348406-25 G, Sigma-Aldrich) solution. A 4 µL droplet of the solution was applied on a 170 µm-thick coverslip (0107052, Marienfeld) and spun to spread the solution across the surface.

### Imaging system

The high-throughput microscope used is Incucyte® S3 Live-Cell Analysis System (Sartorius), which is placed in an incubator and enables imaging over prolonged periods of time. The system capacity enables examination of up to six 96-well plates simultaneously, facilitating the analysis of as many as 576 distinct spheroid samples within a single experiment. Imaging was performed using custom acquisition software. We apply optical modification to a 10X Incucyte objective by the insertion of a Tetrapod phase mask^25,26^, as specified in Opatovski et al.^24^. This objective is referred to as “TP objective” in this work. For comparative imaging, we also use a standard 10X objective, referred to as “ST objective” in this work.

Sample imaging is conducted using wide-field fluorescence and label-free transmitted bright-field imaging. The fluorescence imaging is obtained through the “green channel” which excites GFP. The excitation is in the range of 441–481 nm (blue light), and the emission is in the range of 503–544 nm (green light). In this setup, both illumination and detection are positioned below the sample. In the bright-field channel, both excitation and emission are in the range of 512–538 nm (green light). In this setup, illumination is directed from above the sample, while detection occurs from below.

In each experiment, we typically examined one to four 96-well plates, with one spheroid per well. Multiple conditions were examined, each using several spheroids to ensure reproducibility and enable statistical analysis. For each sample at each time point, we imaged one or two green-channel TP images for z-localization of single fibroblasts or fibroblast clusters, respectively, and one ST bright-field image to track spheroid size and viability. This acquisition resulted in approximately 11.13 s per sample for single fibroblast tracking and 11.63 s per sample for fibroblast cluster imaging. In some experiments, the bright-field image was acquired using the TP objective, enabling substantially faster imaging by eliminating objective switching between samples. Under these conditions, acquisition times were approximately 1.0 s per sample for single fibroblast tracking and 1.58 s per sample for fibroblast cluster imaging.

### Single-shot MLE-based method for 3D single cell localization

Our MLE algorithm for single-shot single cell localization is based on the framework presented in previous work by our group, VIPR^28^ (vectorial implementation of phase retrieval), which enables accurate reconstruction of the system phase mask and computational generation of the PSF at any 3D position. To model cells rather than point sources, we represented each cell as a circular object with an estimated radius of 7 µm. We then simulated the expected cell image by convolving the generated point-source PSF with this circular model. The PSF generator, included in the VIPR framework, takes as input the 3D coordinates and intensity, background level, and reconstructed phase mask to generate the expected image. The phase mask itself is obtained via the VIPR phase retrieval algorithm (using the scalar model), based on a bead z-stack and the optical system parameters. This reconstruction accounts for both the physical phase mask and optical aberrations. Using this forward PSF generation model, we estimate the parameters of the cell, including its z-position, by minimizing the negative log-likelihood loss, comparing the generated image to the experimental one^25,27,28^.

We further perform cell tracking by clustering the resulting 3D localizations across time points using the k-means algorithm^75^. Clustering is performed on the 3D coordinates and time under the assumption that cells exhibit limited movement between consecutive imaging time points.

### Dual-shot depth-from-defocus based method for 3D cell cluster localization

To obtain 3D cell cluster localization, we developed a method based on the depth-from-defocus (DfD) concept, utilizing defocus information encoded by the TP PSF. We use two defocused images of the cluster, acquired below and above the spheroid center, denoted as I₁, below center, and I₂, above center (Fig. 4a–c and Supplementary Note 4). Using a measured bead PSF as our PSF model (Fig. 4d), we estimate the cluster depth by selecting the PSF pair whose convolution with the cluster best matches the observed images. We search among PSF pairs where the axial distance between the two PSFs equals the axial distance between the two imaging planes (see Supplementary Note 3 for details regarding accounting for refractive index mismatch). This axial distance is generally denoted as Δ, and in our settings it is set to 100 µm (Supplementary Note 4).

Our search strategy focuses on identifying, from all PSF pairs that share the distance Δ, the pair that most accurately reconstructs the images of the cell cluster. To achieve this, we apply the Lucy-Richardson deconvolution algorithm^37,38^, using each PSF pair to deconvolve the two cluster images. For the correct PSF pair, both deconvolved images should ideally appear focused. Since we do not have a focused image for comparison, we instead select the PSF pair whose deconvolved images exhibit the highest correlation between them (Fig. 4e, f). For that, the correlation is calculated for each PSF pair and plotted as a function of the candidate cluster depth values, denoted as *z*_cluster_. In our settings, the cluster imaging planes are symmetrically positioned around the central imaging plane (${z}_{{I}_{1}}=-\frac{\Delta }{2}$ and ${z}_{{I}_{2}}=\frac{\Delta }{2}$), so *z*_cluster_ values are obtained for each PSF pair according to:

1 $${z}_{{cluster}}=-n\cdot \left({z}_{{PS}{F}_{1}}+{z}_{{PS}{F}_{2}}\right)/2,$$

where *n* = 1.5 accounts for refractive index mismatch (Supplementary Note 3). The negative of the average is taken because the PSF z-positions describe where the imaging planes are located relative to the object, while the object depth is defined relative to the central imaging plane, resulting in opposite sign conventions. Ultimately, the depth of the cluster is determined as the *z*_cluster_ value that maximizes the correlation (Fig. 4f).

### Statistics and reproducibility

In our high-throughput experiments, replicates were generated for each examined condition. Samples were examined in 96-well plates, each well containing an individual spheroid. Controlled parameters include the number of seeded cancer cells, the ratio of seeded fibroblast cells, ponatinib drug concentration, and cancer cell type. The number of examined samples for each analysis is specified in the corresponding figure legends.

Samples with visible artifacts (e.g., dust and fibers) were excluded from analysis. For the analysis of spheroid-fibroblast interaction patterns, samples for which clusters could not be visually separated were excluded from the analysis of number of formed clusters vs the number of FaDu cells. In addition, samples for which adjacent clusters could not be resolved by computational segmentation were excluded from the analysis of 3D distances between adjacent clusters for different numbers of FaDu cells. For the analysis of drug effect on spheroid-fibroblast interactions, cluster localizations with low localization scores were excluded from the analysis.

Multivariate Hotelling’s *T*^2^ test was used in Fig. 6 as a statistical test to compare each pair of drug concentrations (NT vs low, NT vs high, high vs low). The multivariate distribution consisted of two variables, spheroid diameter and time, or cluster z-depth and time, analyzed separately. The plots indicate the time point at which the differences between the populations become statistically significant (*p* < 0.05).

### Use of large language models (LLMs)

LLMs were used for language polishing and assistance in code writing.

### Reporting summary

Further information on research design is available in the Nature Portfolio Reporting Summary linked to this article.

## Supplementary information

Supplementary Information

Description of Additional Supplementary Files

Supplementary Data 1

Supplementary Video 1

Supplementary Video 2

Reporting Summary

## Data Availability

The source data for the graphs presented in this paper are available in Supplementary Data 1. All other data are available from the corresponding author upon reasonable request.

## Code availability

Code is publicly available at https://github.com/NoamZoref/HTM-LocZ. The version of the code described in this paper is archived at 10.5281/zenodo.20394791^76^.
