# Supplementary material for: Symmetric cancer spheroid-fibroblast organization revealed in 3D by high-throughput microscopy
Source: Commun Biol. 2026 Jul 27;9:1023. doi: 10.1038/s42003-026-10592-3 (PMC13415527; doi:10.1038/s42003-026-10592-3)
Supplement: Supplementary file 1 — Supplementary Information [file 42003_2026_10592_MOESM1_ESM.pdf]

Supplementary Information for

Symmetric cancer spheroid-fibroblast organization revealed in 3D by high-throughput microscopy

Noam Zoref[1,5], Maytal Avrashami[1,5], Nadav Opatovski[2], Paul Keselman[3], Yosi Shamay[1,*], Yoav Shechtman[1,2,4,*]

[1] *Faculty of Biomedical Engineering, Technion - Israel Institute of Technology, Haifa, Israel*
[2] *Russell Berrie Nanotechnology Institute, Technion - Israel Institute of Technology, Haifa, Israel*
[3] *Sartorius Stedim North America Inc., Bohemia, NY, USA*
[4] *Faculty of Electrical and Computer Engineering, Technion - Israel Institute of Technology, Haifa, Israel*
[5] *These authors contributed equally: Noam Zoref, Maytal Avrashami*

**e-mail: yshamay@technion.ac.il; yoavsh@technion.ac.il*

Contents

1. Fibroblast-spheroid interaction patterns: 2D analysis across varying cell ratios........ 3
2. Phototoxicity and photobleaching.. 5
3. Refractive index mismatch effect on depth estimations... 7
4. Imaging and initial analysis.. 9
5. Accuracy estimation of the MLE method for single cell localizations 11
6. Comparison of the MLE method for single cell localizations to localizations using standard imaging 13
7. Accuracy estimation of the DfD method for cell cluster localizations 15
8. Fibroblast-spheroid interaction patterns: 3D analysis across varying cell ratios...... 18
9. Fibroblast interactions with irregularly shaped FaDu spheroids.............................. 19
10. Cal33 interactions with FaDu spheroids 20
11. SK-136 interactions with fibroblasts... 21
12. Assessment of fibroblast detection coverage in early clusters 22
13. Wound healing assay 24
14. Drug effect on spheroid-fibroblast interaction 26
15. FaDu and 3T3 viability assays under ponatinib treatment..................................... 28

1. Fibroblast-spheroid interaction patterns: 2D analysis across varying cell ratios

To investigate the robustness of the spatial interaction pattern between fibroblasts and FaDu spheroids, we systematically varied both the initial number of seeded FaDu cells and the 3T3:FaDu co-culture ratios, with multiple replicates per condition to ensure statistical reliability and reproducibility. FaDu cells were seeded at four different initial amounts: 250, 500, 1000, and 1500 cells per well. After 24 hours, GFP-expressing 3T3 fibroblasts were added at four different ratios relative to FaDu: 1:3, 1:5, 1:10, and 1:20.

Across all tested conditions, fibroblasts consistently self-organized into discrete clusters distributed along the spheroid surface. These "flower-like" arrangements emerged spontaneously within 24 hours of co-culture and were qualitatively preserved across all tested ratios and spheroid sizes, demonstrating the high reproducibility and symmetry of the pattern. Although the 1:20 condition was included in the experimental design, it was excluded from further quantitative analysis due to the low number of fibroblasts, which was insufficient to form a flower-like cluster pattern (Fig. S1a).

To extract the number of formed clusters vs number of FaDu cells (Fig. 1c), we segmented the clusters in the image acquired at the spheroid center, at z=0 μm. Segmentation was performed using OpenCV[1] functions *connectedComponents* and *threshold*. Quantitative analysis revealed that the number of clusters increased with the number of FaDu cells (i.e., spheroid size), while the 3T3:FaDu seeding ratio had minimal influence on the number of clusters formed. However, a small difference was observed for the 1:10 ratio, where the number of formed clusters was slightly higher compared with the other ratios (Fig. S1b).

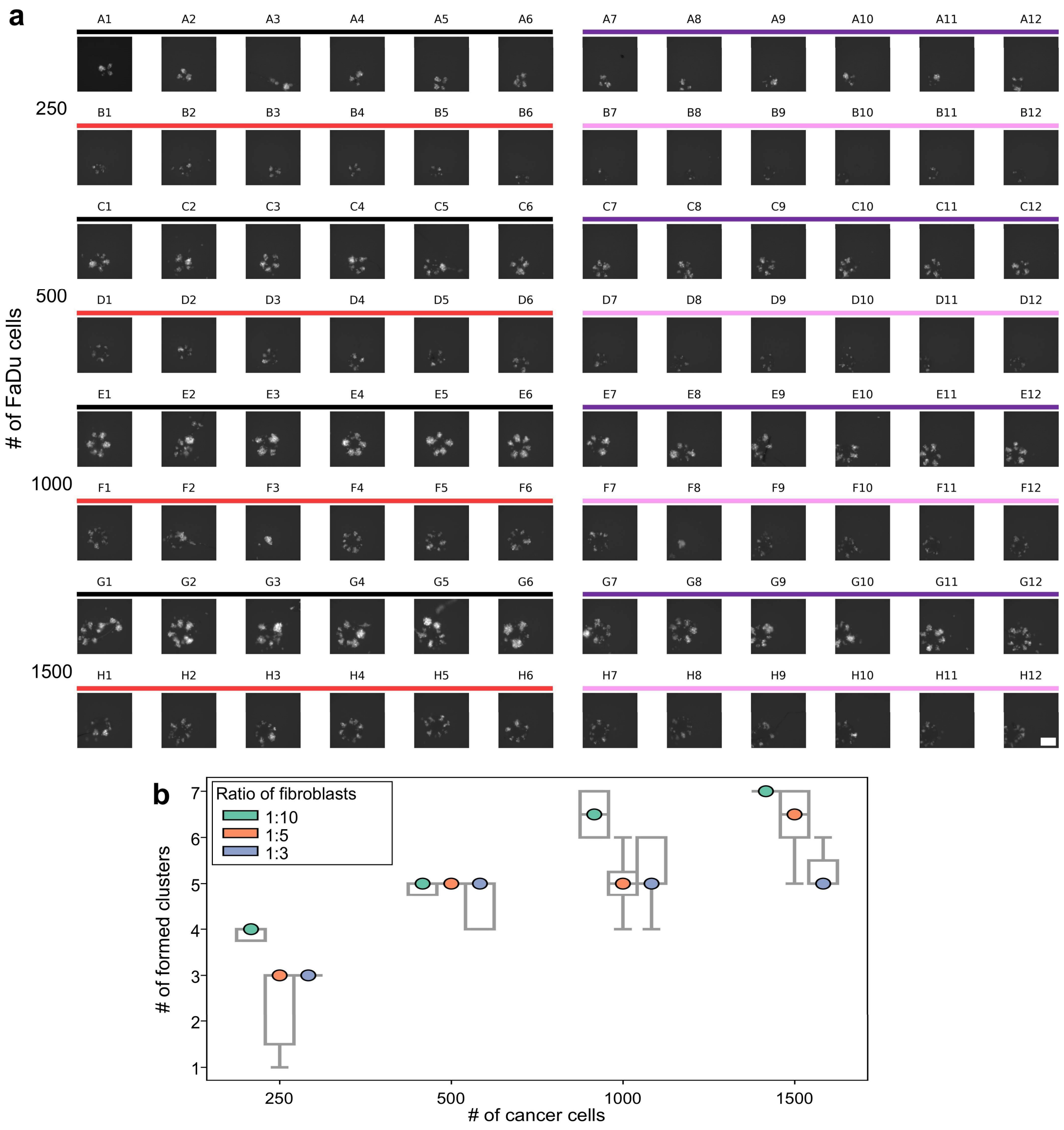

Fig. S1 | 2D analysis of FaDu-3T3 interactions for different amounts of FaDu and 3T3 cells. (**a**) FaDu-3T3 interactions examined using the green fluorescence channel 24 hours after co-culture, across different 3T3:FaDu cell seeding ratios for four initial numbers of seeded FaDu cells, specified in the left column. 3T3:FaDu cell ratios are color-coded as follows: 1:3 (black), 1:5 (purple), 1:10 (red), and 1:20 (pink). White signal indicates 3T3 fluorescence, FaDu cells are not fluorescent. Scale bar: 300 µm. (**b**) Boxplot illustrating the number of formed clusters vs the number of FaDu cells, for different ratios of fibroblasts. Circles indicate median values, colors indicate the ratio of fibroblasts as specified in the legend.

2. Phototoxicity and photobleaching

During our experiments, we observed both photobleaching and phototoxic effects on the cells. The cell lines used were either head and neck cancer (FaDu) or liver cancer (SK-136[2]), co-cultured with green fluorescent protein (GFP) expressing fibroblast cells (3T3). When increasing illumination exposure, either by acquiring more images or shortening the time intervals between acquisitions, we observed phototoxic effects on the fibroblast cells (Fig. S2a-b). This effect is probably a combination of GFP photobleaching and reduced cell viability, which may diminish GFP expression. Notably, our imaging system enabled the acquisition of fewer images compared to standard imaging (single or dual shots for 3D reconstruction), thus mitigating this effect.

We also observed phototoxic effects in the spheroid samples (Fig. S2c). FaDu spheroids exhibited reduced viability at equivalent time points when imaged with a total of 140 acquisitions over 4 days, compared to 86 images, indicating phototoxicity. This was evidenced by morphological changes such as loss of spheroid roundness, poorly defined edges, and single cells visible outside the spheroid outer diameter. A similar trend was observed in SK-136 spheroids on day 2, with reduced viability following 93 image acquisitions compared to 26. As FaDu cells were overall less sensitive to phototoxicity than SK-136 cells, we primarily used them as our cancer spheroid model.

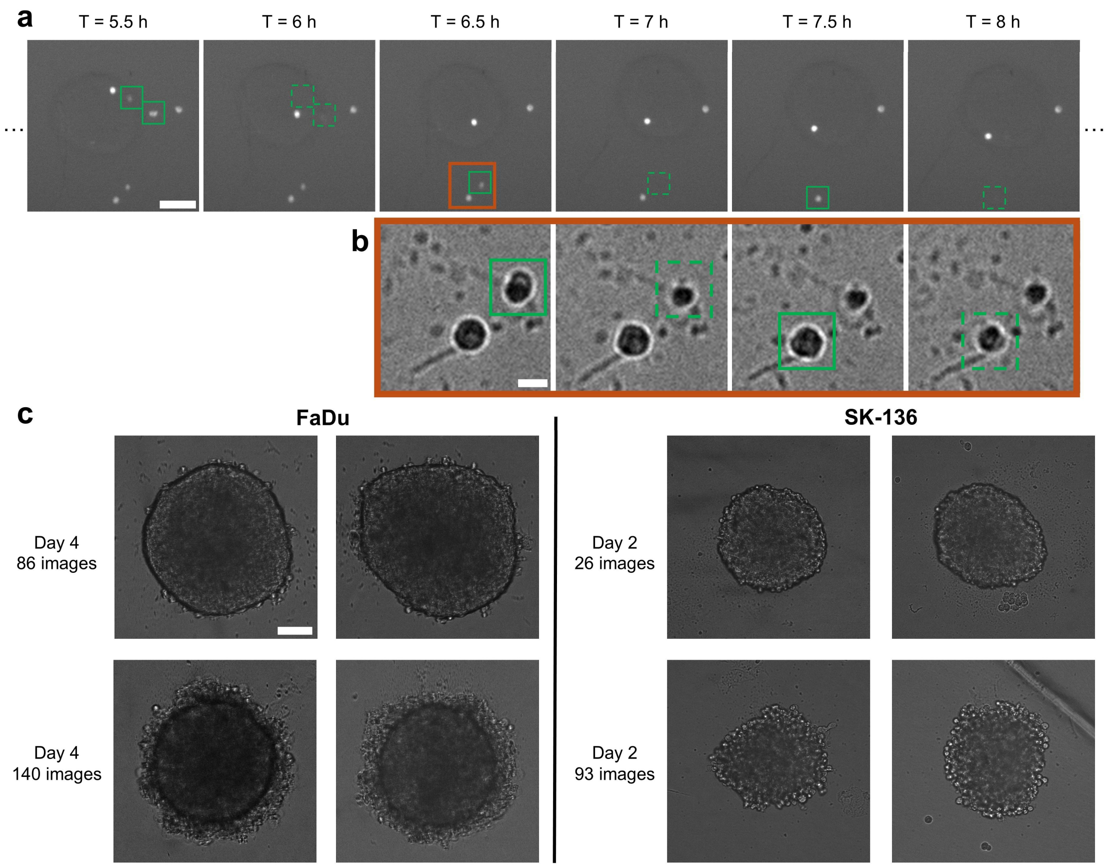

Fig. S2 | Phototoxic and photobleaching effects. (**a**) Phototoxic effects on GFP-expressing fibroblasts interacting with a FaDu spheroid. The timelapse was acquired in the green channel. The sample was imaged 22 times prior to the presented timelapse. The same fibroblast locations are marked across consecutive frames - solid green boxes highlight cells with visible GFP signal, and dashed green boxes indicate where fluorescence has diminished due to phototoxicity and photobleaching. Scale bar: 100 µm. (**b**) Zoom-in on the region highlighted in orange in (a), showing 4 frames (T = 6.5, 7, 7.5, 8 h) acquired using the bright-field channel, indicating that the cells remain present even though they do not appear in the green channel. Green boxes mark the same cell locations, corresponding to those marked in (a). Scale bar: 20 µm. (**c**) Phototoxicity in FaDu and SK-136 spheroids. FaDu spheroid samples (left) were imaged with either 86 (top) or 140 (bottom) images acquired over 4 days. SK-136 spheroid samples (right) were imaged with either 26 (top) or 93 (bottom) images acquired over 2 days. Scale bar: 100 µm.

3. Refractive index mismatch effect on depth estimations

Imaging in our experiments is performed using a 10X air objective ($n_{obj} = 1$), either in an ST configuration or integrated with a TP phase mask. All spheroid samples are embedded in medium with a refractive index of $n_{medium} \approx 1.34$, leading to a refractive index mismatch between the objective and the sample, which introduces axial distortion. Additionally, the spheroids are positioned in round-bottom, ultra-low attachment (ULA) wells (Fig. 1a), introducing further axial distortion.

To estimate the effects of index mismatch, we examined two sample configurations. The first sample consists of beads adhered to 170-µm thick glass coverslips, separated by a ~250-µm thick layer of either medium or air (Fig. S3a). This sample is intended to help approximate the index mismatch effect introduced by the medium. The second sample consists of a single nuclear-stained spheroid embedded in medium and positioned in our round-shape well, thus incorporating both the medium and well contributions to index mismatch effects. Both samples demonstrate that the index mismatch in our system causes an axial "shrinking" effect, where the nominal focus position (NFP), defined by the objective axial position, is smaller than the actual focus position (AFP), which corresponds to the true axial position within the sample. This is due to n_{obj} (air) being lower than n_{sample}, an effective refractive index reflecting contributions from both the medium and the well or coverslip (Fig. S3a-b)[3,4]. The bead sample shows a factor of 1.412≈1.4, while the spheroid sample shows a factor of 1.511≈1.5, assuming a spherical sample geometry. Both approximately follow the relation $NFP = \frac{n_{sample}}{n_{obj}} AFP$, as described by Hell et al[4]. Based on this analysis, we approximate the index mismatch effect using a factor of 1.5, which reflects the spheroid-in-well configuration and more closely matches our imaging conditions. A more accurate correction would require specific calibration experiments.

Next, we aimed to estimate the axial elongation of the PSF in bead samples embedded in medium vs air. This analysis is relevant for our algorithms, which use bead-in-air PSF as a model for the PSF introduced for cells embedded in medium within round-bottom wells. Under our imaging conditions, the PSFs obtained from bead-in-air and bead-in-medium samples appear nearly identical in axial extent (Fig. S3c). While PSF broadening due to index mismatch has been reported in previous studies[3,5], our configuration, i.e. air objective imaging of a sample in liquid media, may lead to an approximate counterbalancing between this broadening and the axial "shrinking" caused by refractive index mismatch, resulting in similar PSFs for the same objective movement. While the sample holder differs in the spheroid experiments, our algorithmic analysis supports that this approximation of a nearly unchanged PSF axial profile for a given objective movement still holds when applied to cells embedded in medium within round-bottom wells (Supplementary Notes 5 and 7).

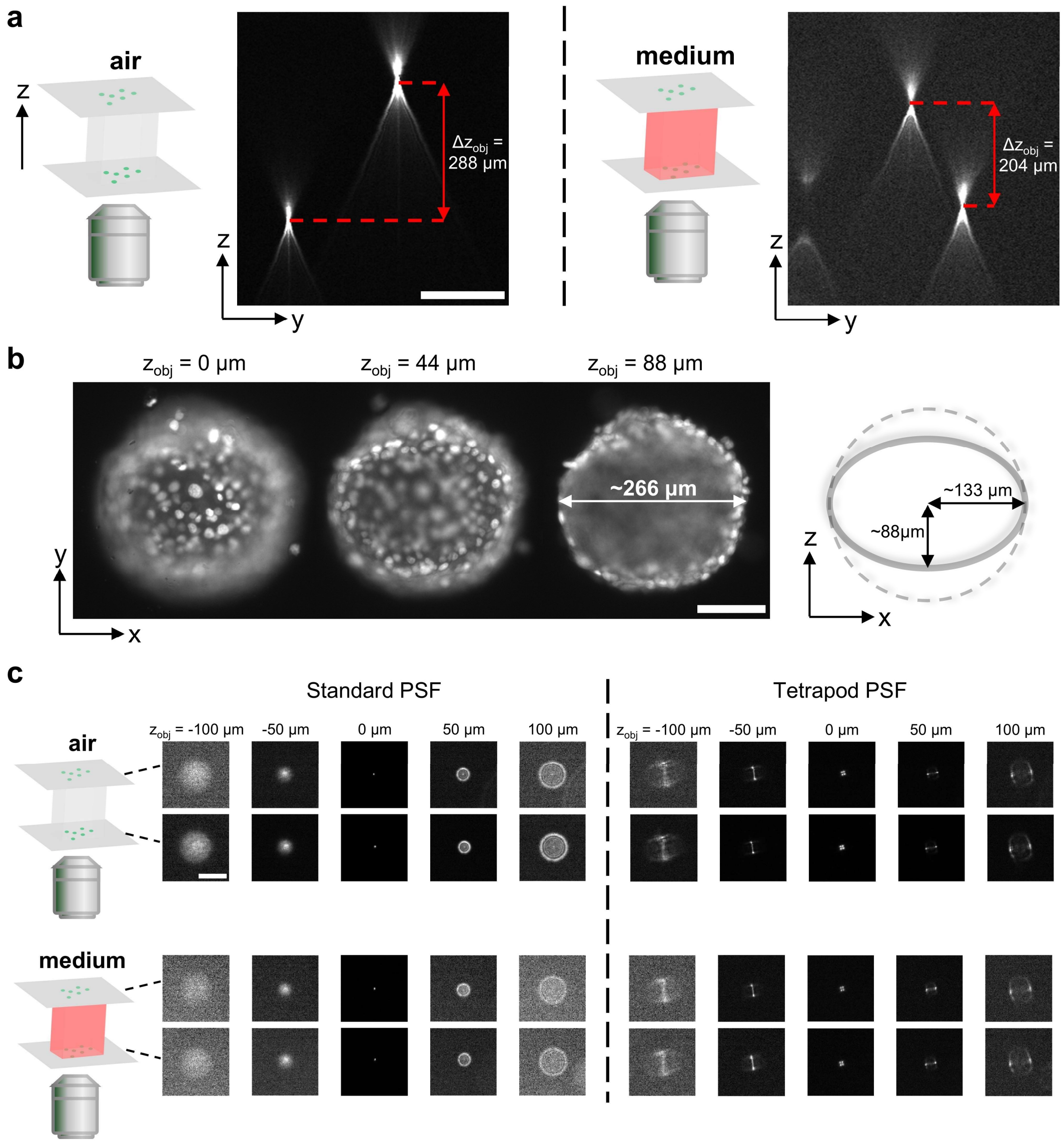

Fig. S3 | Experimental estimation of refractive index mismatch effect. (**a**) Bead-on-coverslip samples linked via an adhesive frame with a central cavity (25 µl Gene Frame, Thermo Fisher Scientific, AB0576), imaged under two different immersion conditions. In the first condition (left), the cavity is left empty (air-filled), and the observed shift in focal planes due to objective movement is $\Delta z_{obj} = 288\ \mu m$. In the second condition (right), the cavity is filled with medium, resulting in a reduced focal shift of $\Delta z_{obj} = 204\ \mu m$. Scale bar (y-axis): 100 µm. (**b**) Left: z-stack of a nuclear-stained spheroid, obtained from Opatovski et al.[6] and imaged using the same system in the ST configuration. Right: Corresponding illustration of the refractive index mismatch effect – dashed line represents expected round spheroid form, and the solid line marks the resulting index mismatch effect-induced "shrunken" form. Scale bar: 100 µm. (**c**) PSFs of the bead samples presented in (a), showing a single bead PSF, from either the upper or bottom bead sample, imaged with both ST and TP objectives, under both air and medium immersion conditions. Scale bar: 50 µm.

4. Imaging and initial analysis

High-throughput 3D tracking of spheroid-fibroblast interactions consists of time-lapse imaging of the samples. This process involves both a data acquisition method and an analysis framework. For data acquisition, samples are scanned using custom acquisition software, which allows selection of the z-slices to image, the objective to use, and the imaging time interval.

For each experiment, we performed two preliminary steps. The first was Tetrapod (TP) PSF model estimation, obtained by capturing a z-stack of a green fluorescent bead. The second step involved estimating the axial center of each spheroid using a z-stack acquired in the bright-field channel for each sample in the experiment. In this initial scan, the axial distance between consecutive frames was 8 μm, and 50 images per well were acquired. The axial center for each well was determined using the Laplacian operator, applied twice to all images after cropping them to 700×700 pixels around their approximate lateral center. The norm of each resulting image was calculated, and the center z-position for each well z-scan was identified as the image with the highest norm. The determined center z-position for each well was then set as $z = 0$ for that well for the rest of our experiment. This step was essential to account for plate inhomogeneities that caused shifts in z of up to 400 μm and variability in spheroid sizes.

Subsequent imaging for each well and time point includes the acquisition of one or two green-channel TP images for z-localization of fluorescent cells or cell clusters, respectively, and one standard (ST) bright-field image to estimate spheroid size and viability. In some experiments the bright-field image was acquired using the TP objective. The analysis framework consists of a multi-step process to obtain accurate high-throughput 3D information.

The initial analysis step involves extracting the spheroid 2D location and diameter based on bright-field imaging for each well. This step is performed on a single image acquired at the beginning of the imaging session, centered at the predefined spheroid axial center. The spheroid is localized by the image Laplacian. We apply Otsu thresholding[7] and mean averaging to the Laplacian image to enhance contrast between the low-intensity, label-free spheroid and the surrounding background. We then use OpenCV[1] *distanceTransform* to obtain the spheroid diameter and lateral center location.

In the second step, we generate time-lapse stacks of green-channel TP images for each spheroid. For maximum-likelihood estimation (MLE) localizations of single cells, a single image is acquired per time point. For depth-from-defocus (DfD) localizations of cell clusters, two images are acquired at each time point, resulting in two parallel time-lapse stacks. The images are cropped and stacked based on the 2D locations obtained in the initial step. The images are then aligned to account for the spheroid movement between frames, which occurs mostly in the lateral plane. For DfD analysis, alignment is performed on only one of the two time-lapse stacks (i.e., one z-plane), and the same transformation matrices are then applied to the second stack. The 2D alignment matrices, which register each

spheroid image to the previous one, are calculated using the python package *pyStackReg*[8] (https://pypi.org/project/pystackreg/).

In the third step, we localize cells and cell clusters. 2D localizations are performed in each frame using the python package skimage[9] function *peak_local_max*, which detects intensity peaks in images. The detected localizations are then filtered based on intensity relative to a defined percentile of the image intensity. Adjustable parameters include the minimum distance between localizations, the intensity percentile threshold, and parameters for Gaussian blurring applied to the image prior to localization. The blurring is applied to mitigate the dual-peak effect introduced by the TP PSF convolution, which is particularly evident for small objects. The obtained 2D locations are then transformed using the registration matrices calculated in step two, ensuring correspondence with the aligned time-lapse stack. Detected localizations are subsequently cropped to a specified size from the time-lapse stack or both stacks in the case of DfD analysis. Next, we perform z-localizations for each detected cell or cell cluster using the MLE-based or the DfD-based method, respectively.

5. Accuracy estimation of the MLE method for single cell localizations

To estimate the accuracy of our single cell localization method, which is based on maximum-likelihood estimation (MLE), we compared MLE z-localizations against ground truth z-localizations. To acquire such a dataset, we imaged single fibroblast interactions with spheroids in the green channel, using both ST and TP objectives. In this experiment, fibroblasts were added in low concentration (approximately 10 cells) to 36 samples of spheroids in medium. For each sample we imaged two z-stacks – the first using the TP objective, consisting of 4 images with $\Delta z_{TP} = 50$ µm, and the second using the ST objective, consisting of 60 images with $\Delta z_{ST} = 2$ µm.

The MLE-based method for single cell localization was used to obtain localizations from the TP images, following the steps depicted in Supplementary Note 4. The ground truth localizations were obtained from the ST z-stack, by cropping each cell z-stack in xy, generating a yz projection, and detecting the z-value of maximum intensity using peak detection (Fig. S4a). Overall, we obtained 291 localization pairs (filtering out 17 low-correlation outliers) used in our analysis.

The overall localization accuracy was estimated by computing the median error between the estimated localizations and the ground truth localizations, which was 7.6 µm. Additionally, scanning with $\Delta z_{ST} = 2$ (corresponding to a 3 µm axial distance in the sample; see Supplementary Note 3) introduced a 1.5 µm error (half the inter-frame distance), resulting in a total accuracy estimate for the MLE algorithm of ~9 µm. The close alignment of our estimated z vs ground-truth z scatter plot to the identity line (y=x) further supports our use of the bead-on-coverslip PSF, without axial scaling, as our approximated PSF model (Supplementary Note 3 and Fig. S4b). We further analyzed the dependence of the distance between the ground-truth and the focal plane on the localization accuracy. Fig. S4c shows that the error increases when the ground-truth z is close to the focal plane. The likely reason is that when the ground-truth z is near the focal plane, the cell TP image changes only slightly with z because it results from the convolution of a nearly focused TP PSF with a circle, making it difficult to determine the exact z location. The error also increases when the focal plane is too far from the ground truth z, probably due to strong blur and low SNR. This is particularly evident at TP z slices of 50 µm and 0 µm.

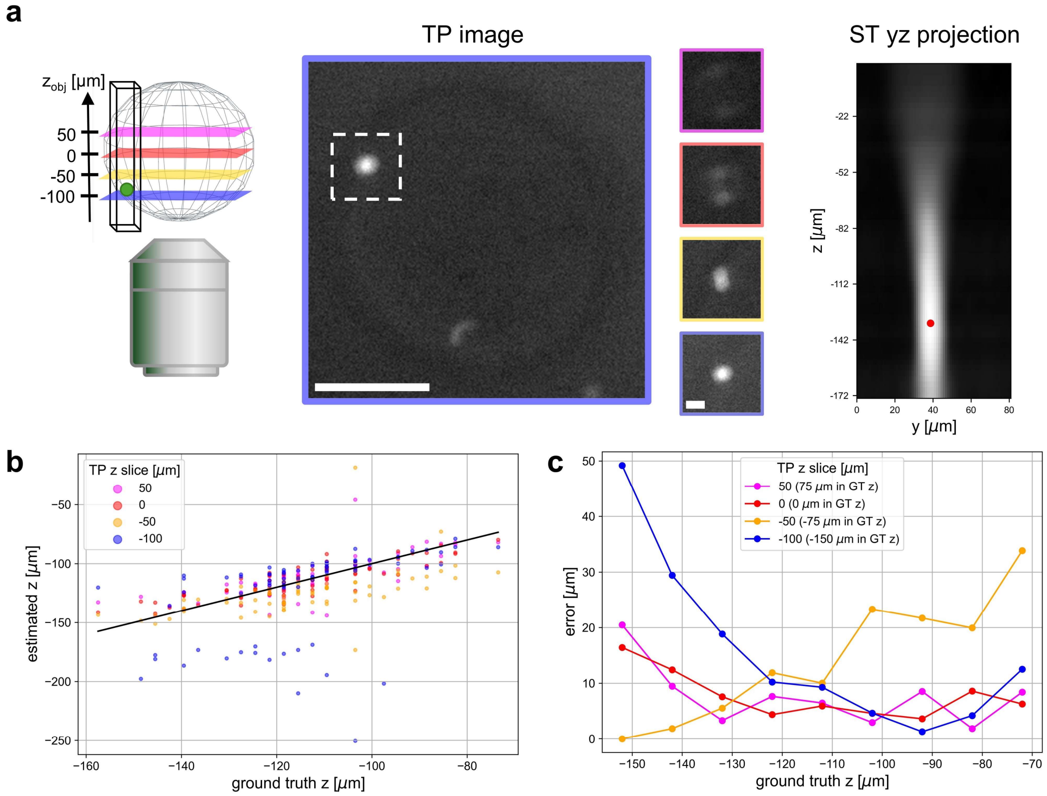

Fig. S4 | MLE single cell localization accuracy estimation. (**a**) Single fibroblast interaction with a FaDu spheroid. Left to right: Illustration of the sample with imaged TP z-slices, specified by objective z-positions. Blue, yellow, red and magenta indicate z = -100, -50, 0, and 50 µm, respectively; TP image of the fibroblast-spheroid sample focused on z = -100 µm. Scale bar: 100 µm; Zoom-ins of the cell marked with a white dashed box in the TP image, shown for different z values which are indicated by the corresponding color. Scale bar: 20 µm; YZ projection of a z-stack of the cell, acquired using the ST objective. The obtained ground truth z-localization is marked with a red dot at z = -133.5 µm. (**b**) Comparison between estimated z and ground truth z for different imaged TP z-slices. The black line represents the identity line, y=x, highlighting the obtained error. (**c**) Error for ground truth estimations for different TP z-slices. The legend specifies the corresponding ground truth (GT) z for each TP z-slice defined by the objective z-position (Supplementary Note 3).

6. Comparison of the MLE method for single cell localizations to localizations using standard imaging

To compare our MLE method using TP images against localizations that can be obtained using standard imaging, we performed localizations using standard imaging using two approaches. The dataset used here is the one used in Supplementary Note 5.

Firstly, to obtain localizations with standard (ST) PSF using the same amount of data as used in our MLE method for TP images, meaning a single image per time point, we executed our MLE method with standard PSF. Examples of the model estimations are shown in Fig. S5a. We obtained localizations for each cell using three different ST z-slices (-101, -51 and -1 μm, which correspond to -151.5, -76.5 and -1.5 μm in GT z; see Supplementary Note 3). These localizations were compared against ground truth localizations obtained from the dense ST z-stack, as explained in Supplementary Note 5. After filtering out low-correlation outliers using the same threshold used for TP estimations in Supplementary Note 5, only 78 out of 195 localizations remained. A large number of localizations received a low score due to the limited depth encoding of the ST PSF (out-of-focus PSFs look similar in both z directions) and their low SNR far from focus (since the depth-of-field of the ST PSF is significantly smaller than that of the TP PSF). These localizations resulted in a median error of 9.2 μm between the estimated localizations and the ground truth localizations. Additionally, as in Supplementary Note 5, scanning with $\Delta z = 2$ μm (which corresponds to a 3 μm axial distance in the sample) introduced an additional 1.5 μm error (half the inter-frame distance). Overall, the obtained accuracy was ~11 μm, slightly worse than the ~9 μm accuracy achieved with the MLE method for TP images, and obtained with substantially fewer localizations remaining after threshold filtering. When raising the threshold, 161 localizations remained and resulted in an overall accuracy of ~15 μm (Fig. S5b).

Secondly, we obtained localizations from a standard z-stack, with different sampling densities, using a Δz of 10, 20 and 30 μm. The z-stacks were linearly interpolated to achieve $\Delta z = 2$ μm, as used in Supplementary Note 5. The localization accuracy for each sampling density was estimated as the median error between the estimated localizations from the sparse z-stack and the ground truth localizations with $\Delta z = 2$ μm. The accuracies were 3, 3 and 9 μm for Δz of 10, 20 and 30 μm, correspondingly (Fig. S5c). Overall, with the introduced additional 1.5 μm error, the obtained accuracies were 4.5, 4.5 and 10.5 μm for Δz of 10, 20 and 30 μm, correspondingly. This analysis indicates that achieving the ~9 μm single-shot MLE accuracy obtained with the TP PSF (Supplementary Note 5) with ST z-stack acquisitions requires axial sampling of $\Delta z < 30$ μm (which corresponds to $\Delta z < 45$ μm in the sample), resulting in at least 3–6-fold increase in the number of acquired images.

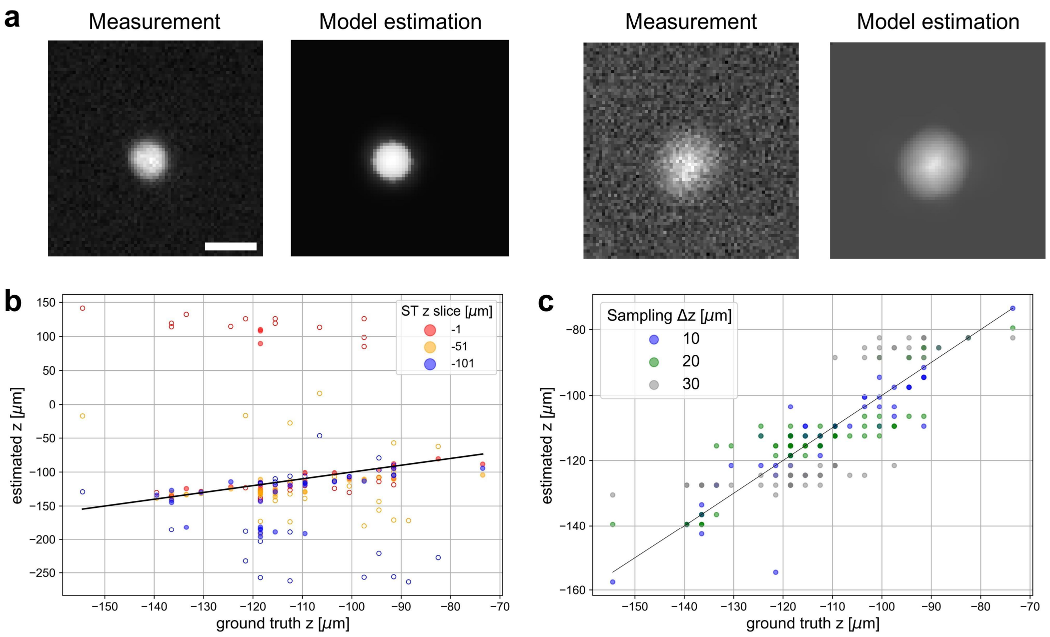

Fig. S5 | Single cell localizations using standard imaging. (**a**) Cell ST measured images and their corresponding ST model estimations using the MLE method. The z values yielded by the model, from left to right, are -141.3 μm and -138 μm, for imaged ST z-slices of -101 and -51, respectively. Scale bar: 20 μm. (**b**) Comparison between estimated z and ground truth z for different imaged ST z-slices. The black line represents the identity line, y=x, highlighting the obtained error. Hollow markers represent localizations that were filtered out due to low correlation. (**c**) Comparison between estimated z and ground truth z for localizations obtained using ST z-stacks with different sampling densities, specified in the legend. The black line represents the identity line, y=x, highlighting the obtained error for each sampling density.

7. Accuracy estimation of the DfD method for cell cluster localizations

To estimate the accuracy of our depth-from-defocus (DfD) cell cluster localization method, we compared DfD z-localizations against ground truth z-localizations. To generate a dataset representative of spheroid interaction with fibroblast clusters across different stages of cluster formation, we added fibroblasts to the wells twice, with 24 hours between the additions. In this experiment, spheroids were formed by seeding 500 SK-136 cells per well. Fibroblasts were first added 6 hours afterwards. Following the second fibroblast addition, samples were imaged in the green channel, using both ST and TP objectives. For each objective a z-stack was acquired – for the TP objective, we acquired 3 images centered at the spheroid axial center with Δz_{TP} = 50 μm, and for the ST objective we acquired 51 images with Δz_{ST} = 4 μm.

The DfD method for cell cluster localization was used to obtain localizations from the TP images, following the steps depicted in Supplementary Note 4. We followed this procedure for each pair of TP slices, to compare the accuracy of the different options. The ground truth localizations were obtained from the ST z-stack using the same procedure as in the MLE accuracy evaluation (Supplementary Note 5 and Fig. S6a). Overall, 213 fibroblast cluster z-localizations were obtained for both ST and TP methods.

Several factors can introduce biases in cluster z calculations for both ST and TP methods. Firstly, both methods base their z calculations on the highest intensity, creating a bias towards the brighter areas in the cluster, which do not always correspond to the cluster center. Moreover, for large clusters, we expect both z calculations to be lower than the actual z, since the cluster upper parts can appear dimmer due to scattering. Additionally, light scattering by the dense cell structure of spheroids may further affect perceived z-values, particularly for clusters located deeper within the spheroid.

To detect the pair of TP slices yielding the highest z-localization accuracy, we compared ST ground truth z to TP estimated z for the three TP pair combinations of the imaged slices (z = -50, 0 and 50 μm), referred to as [0,50], [-50,0], and [-50,50] (Fig. S6b), where z = 0 denotes the spheroid axial center and z values indicate objective positions. After filtering for correlation scores greater than 0.8 and removing localizations at the edges of the examined range, we obtained 183, 183 and 192 localizations for [0,50], [-50,0], and [-50,50], respectively. Across all three options, we observed a similar alignment with the identity line (y=x), indicating a match between the ground truth z and the estimated z and further supporting our approximated PSF model (Supplementary Notes 3 and 5). However, for both [0,50] and [-50,0] configurations the extrapolation beyond the range between the imaged z-slices is less aligned with the identity line, resulting in less accurate z-localizations (Fig. S6b). Subsequent calculation of the median absolute errors yielded comparable values of 12, 12, and 9 μm for the configurations [0,50], [-50,0], and [-50,50], respectively (Fig. S6c).

Further analysis of the median absolute errors across these configurations, considering different ground truth z margins, aimed to quantify each configuration extrapolation capability beyond the range between the imaged z slices. The examined ground truth z

margins were [0,50] and [–50,0], defined by objective positions and corresponding, respectively, to axial ranges of [0,75] and [–75,0] within the sample (Supplementary Note 3). The findings indicated that while the [-50,50] configuration demonstrated efficacy across both ground truth z regions (with a median value of 9 μm for both ground truth z margins [0,50] and [-50,0]), the other configurations exhibited higher median values beyond their respective regions (Fig. S6d).

Based on the previous analysis, we chose to perform our experiments with the [-50,50] z-slice configuration, imaging each sample at z = -50 μm and z = 50 μm. The overall z-localization accuracy for the chosen configuration was estimated as the median absolute error between the estimated and ground-truth z-localizations, which was 9 μm. Additionally, scanning with $\Delta z_{ST} = 4$ (corresponding to a 6 μm axial distance in the sample) introduced an error estimated as half the inter-frame spacing (3 μm), resulting in a total accuracy estimate of 12 μm for the DfD algorithm.

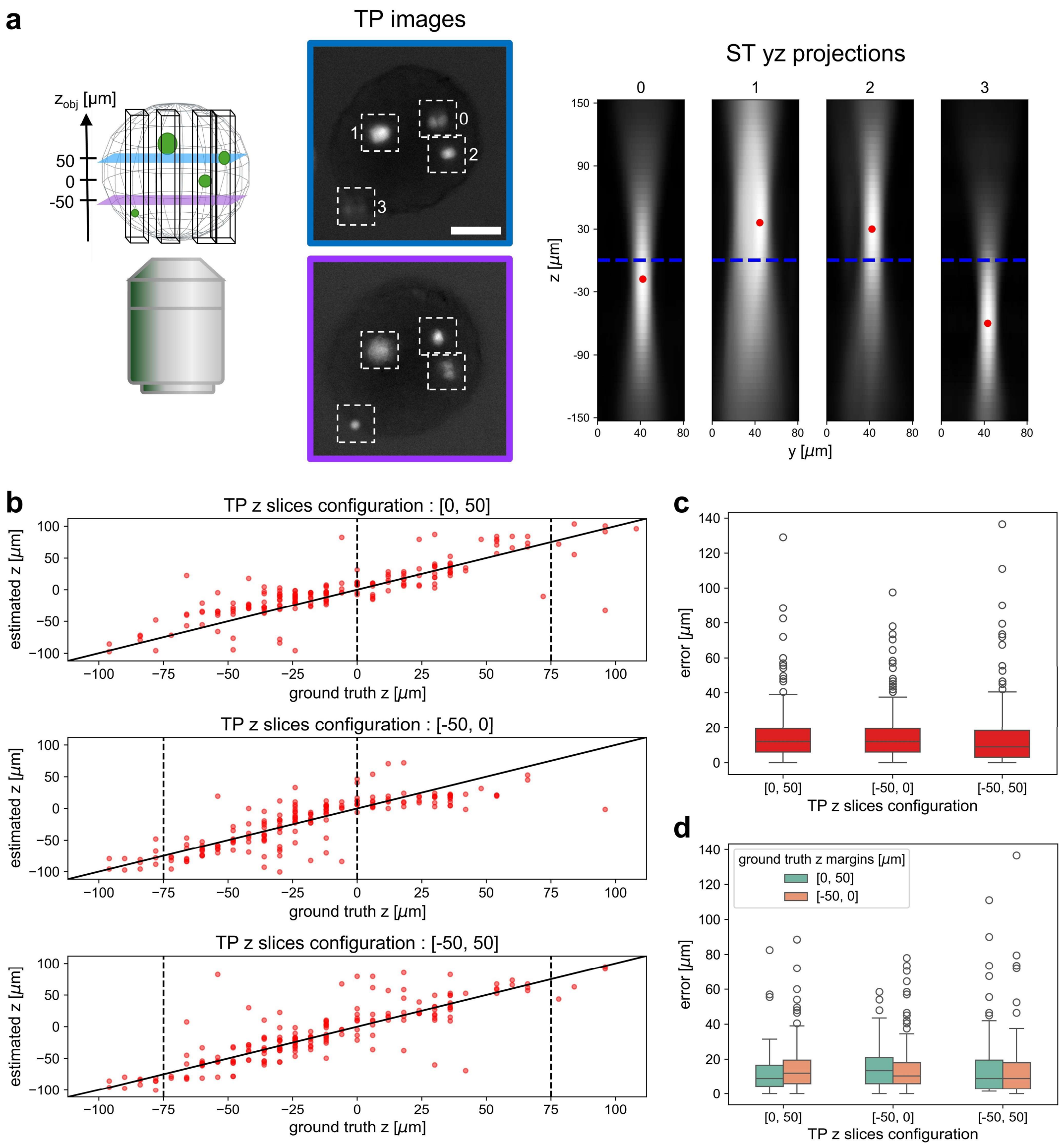

Fig. S6 | DfD localization accuracy estimation. (**a**) Fibroblast cluster interaction with a SK-136 spheroid. Left to right: Illustration of the sample with imaged TP z-slices, specified by objective z-positions. Purple and blue indicate z = -50 and 50 μm, respectively; TP images of the sample focused on different z values, indicated by the corresponding color. Examined clusters are numbered and marked in white dashed boxes. Scale bar: 100 μm; YZ projection of ST z-stacks of the clusters marked in the TP images. Upper row indicates the corresponding cluster number. The obtained ground truth z-localizations are marked with red dots, and the blue dashed line marks z=0. (**b**) Estimated z vs ground truth z, compared to the identity line (y=x, black line), for different z-slice configurations: [0,50], [-50,0] and [-50,50]. Dashed black lines mark the boundaries of the axial ranges within the sample corresponding to each configuration range (Supplementary Note 3). (**c**) Boxplot analysis representing absolute error per z-slice configuration, for the data presented in (b). (**d**) Boxplot analysis representing absolute error per z-slice configuration, for two ground truth z margins, [0,50] and [-50,0] (defined by objective positions).

8. Fibroblast-spheroid interaction patterns: 3D analysis across varying cell ratios

To perform the 3D analysis and 3D reconstruction of the "flower-like" pattern (Fig. 5), we segmented the clusters in the TP image acquired at the spheroid center, at z=0 µm. Segmentation was performed using OpenCV[1] functions *connectedComponents* and *threshold*, as in Supplementary Note 1. For each cluster, we extracted certain parameters including total area, 2D weighted lateral center and z-location: Cluster total areas were calculated by masking each cluster; 2D weighted lateral centers were calculated using a multiplication of each cluster mask with the TP image; z-locations were obtained by cropping each cluster by its lateral center in two z-slices (z=±50 µm) and running the DfD algorithm. To generate the 3D reconstructions with approximate cluster sizes (Fig. 5a,c), we utilized the cluster area information in addition to 3D locations. 3D spherical distances were estimated by calculating the length of the arc connecting the 3D cluster centers, for an estimated spheroid radius (Fig. 5b). Visualization of these arcs is shown in Fig. 5a. Further analysis of the 3D spherical distances between adjacent clusters showed a consistent spacing, independent of FaDu cell number or fibroblast ratio, except for 1:10 ratio, that presented a corresponding slightly shorter distance between clusters (Fig. S7).

To reconstruct in 3D the generation process of the flower-like pattern presented in Fig. 5c, we followed the steps depicted in Supplementary Note 4, including 2D localization of clusters or cells, their cropping in two z planes and 3D localization using the DfD algorithm. Analysis of the images showed a trend of rising cells, as shown in Fig. 3 for single cells. Therefore, to perform z-localizations with higher accuracy, we used different z-slices for different time points, matching the approximated z-locations (Supplementary Note 7) – for T=16 h we used the images focused on z=-50 µm and z=50 µm, while for T<16 h we used the images focused on z=-100 µm and z=0 µm. We then performed manual filtering of images with low SNR and wrong localizations.

These findings highlight the reproducibility of the self-organized interaction pattern and demonstrate the suitability of our high-throughput platform for capturing and analyzing complex, spatially coordinated cellular behaviors across diverse conditions.

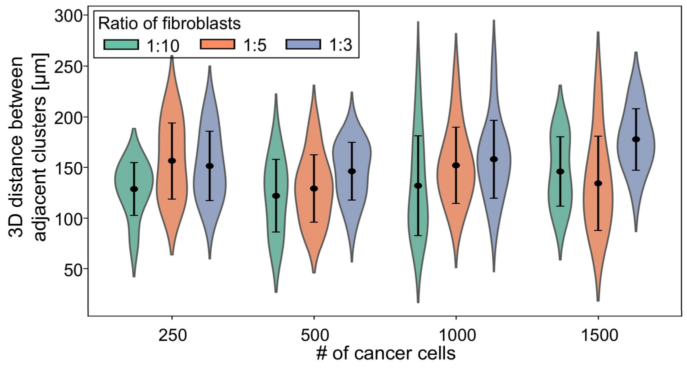

Fig. S7 | 3D spherical distance analysis of FaDu-3T3 interactions for different amounts of FaDu and 3T3 cells. Violin plot showing mean 3D spherical distances between adjacent clusters for different numbers of FaDu cells and fibroblast ratios. Colors indicate the ratio of fibroblasts as specified in the legend.

9. Fibroblast interactions with irregularly shaped FaDu spheroids

We generated irregularly shaped spheroids by seeding $1{\cdot}10^6$ FaDu cells in ultra-low attachment (ULA) flasks. After spheroid formation (24 hours), $3.3{\cdot}10^5$ 3T3 cells were added to the flask and were allowed to penetrate the spheroids over an additional 24-hour incubation period. Unlike round-bottom ULA plates, ULA flasks facilitate the formation of multiple spheroids of varying sizes, with fibroblasts likely spreading homogeneously throughout. Spheroids were then imaged using Incucyte S3, after transferring 150µL from the flask to a flat-bottom 96-well plate. Fibroblasts were observed to infiltrate the irregularly shaped spheroid as clusters, rather than as single cells, exhibiting a pattern similar to the pattern observed in round-shaped spheroids (Fig. S8).

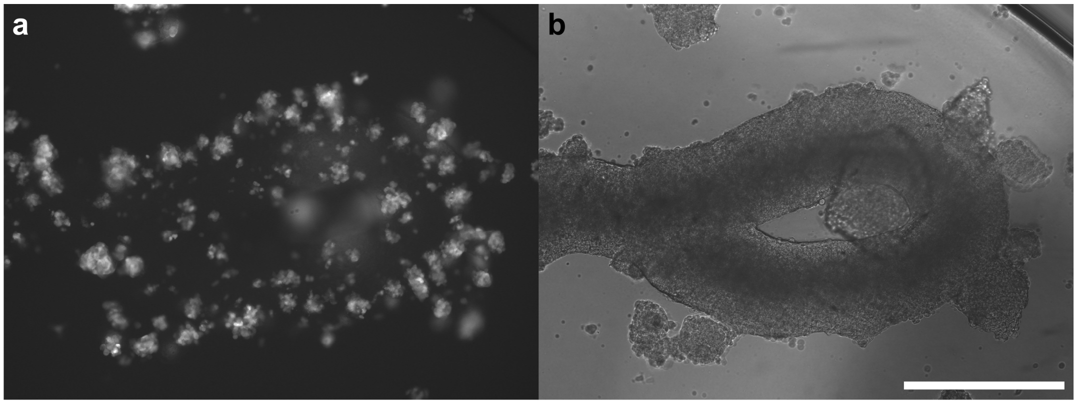

Fig. S8 | Fibroblast organization in asymmetric spheroids. Images of asymmetric spheroid-fibroblast interactions acquired using the ST objective and focused on the spheroid center. (**a**) Fluorescence image, acquired in the green channel. White signal indicates 3T3 fluorescence, FaDu cells are not fluorescent. (**b**) Image acquired using the bright-field channel. Scale bar: 500 µm.

10. Cal33 interactions with FaDu spheroids

To assess whether the cluster penetration pattern observed with fibroblasts and FaDu spheroids is cell-type specific, we conducted a parallel experiment in which we exchanged the fibroblasts with GFP-expressing Cal33 cells - a head and neck squamous cell carcinoma derived from the tongue, in contrast to FaDu cells, which originate from the hypopharynx. FaDu cells were seeded at varying numbers of cells and allowed to form spheroids over 24 hours. Cal33 cells were then added at different ratios and co-cultured for an additional 24 hours. The observed interaction pattern over a few days showed a relatively uniform distribution of cells around the spheroid axial center (Fig. S9), in contrast to fibroblasts, which formed discrete, approximately equally spaced clusters (Fig. 1 and Fig. 5), suggesting that the infiltration pattern observed for fibroblasts is cell-type specific.

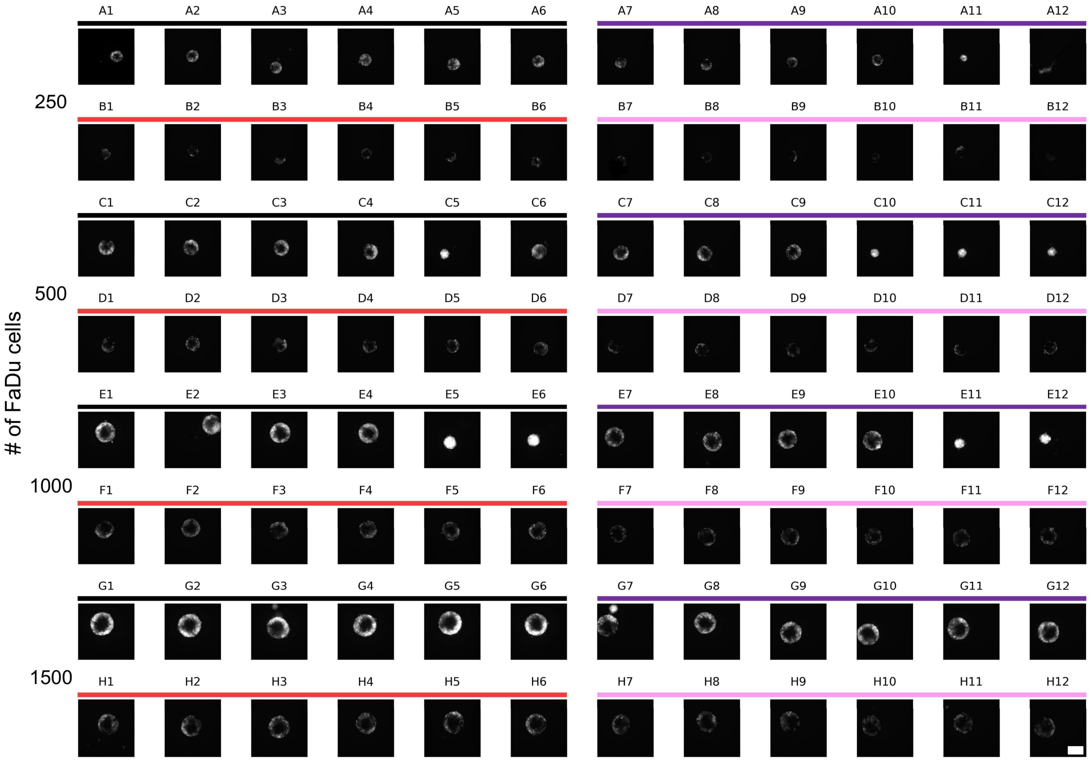

Fig. S9 | Cal33 cell interactions with FaDu spheroids. The interactions were examined using the green fluorescence channel 24 hours after co-culture, across different Cal33:FaDu cell seeding ratios for four initial numbers of seeded FaDu cells, specified in the left column. Cal33:FaDu cell ratios are color-coded as follows: 1:3 (black), 1:5 (purple), 1:10 (red), and 1:20 (pink). White signal indicates Cal33 fluorescence, FaDu cells are not fluorescent. Scale bar: 300 µm.

11. SK-136 interactions with fibroblasts

We investigated the interaction of fibroblasts with SK-136 spheroids to evaluate whether a different cancer cell type, beyond FaDu, exhibits a similar interaction pattern. Fibroblasts were added to 24-hour-old SK-136 spheroids, following the same protocol used for FaDu spheroids (Fig. 1). In contrast to the symmetrical cluster pattern observed for FaDu, fibroblasts with SK-136 spheroids formed less symmetrical patterns and appeared to penetrate more deeply while maintaining rounded cluster morphology (Fig. S10), rather than the elongated structures seen for FaDu after 48 hours (Fig. 1b). In both cases, during the early stages of spheroid development (up to 72 hours), the extracellular matrix (ECM) barriers may not yet be fully formed, allowing easier initial penetration. However, at later stages, SK-136 spheroids were found to be less permeable than FaDu spheroids[10], potentially affecting fibroblast migration and distribution within the spheroid.

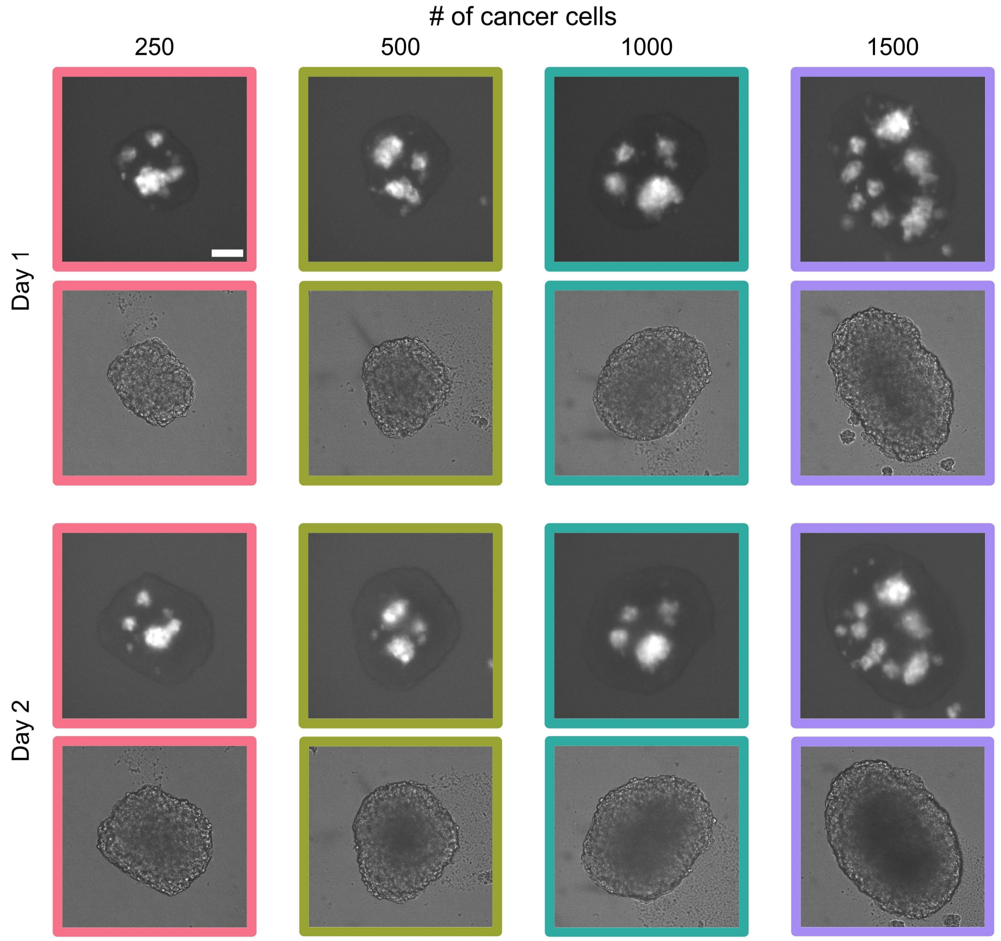

Fig. S10 | SK-136 interaction pattern with fibroblasts. Fibroblast cluster formation in SK-136 spheroids. Fibroblasts were added 24 hours after seeding SK-136 cells to form spheroids composed of 250, 500, 1000, or 1500 cells, as described in Fig. 1. The concentrations of the fibroblasts added were 1:3 for each amount of seeded SK-136 cells. Interactions were examined on days 1 and 2. For each day, the top row shows fluorescence images, and the bottom row shows bright-field images. Scale bar: 100 µm.

12. Assessment of fibroblast detection coverage in early clusters

To verify that most of the fibroblasts are imaged and not occluded by the spheroid, we imaged their gathering around the spheroid. Fibroblast gathering shows a similar pattern across all our samples. Fig. S11 shows one representative sample in which fibroblasts were counted over the entire field of view (FOV). Fluorescent cell counting was performed using the ImageJ plugin TrackMate[11,12]. The detector used was the Laplacian of Gaussians, with a manually adjusted estimated object diameter of 9 pixels and a quality threshold of 1. In this experiment, approximately 200 fibroblasts were seeded manually.

The images show fibroblasts as they approach the spheroid. Imaging starts when only around half of the fibroblasts have reached the spheroid area. Later, we detect most of them, until the fibroblasts arrange in dense clusters, so the algorithm cannot be used to segment them individually. Errors in segmentation occur for cells with low signal, for out-of-focus cells showing evident TP shape that is detected as two cells, and for cells in clusters.

Overall, this analysis shows that the vast majority of seeded cells are imaged and not occluded. The number of detected cells is close to the intended seeding density (approximately 200 cells), with a small discrepancy that falls within the expected variability associated with manual cell seeding and image-based counting, which is commonly reported to be on the order of 5–15%. Secondly, the images show that fibroblasts tend to reach the spheroid from the sides rather than from above and to gather around its bottom half. If some fibroblasts reach the upper part of the spheroid, they may be occluded. However, the images suggest that, if this occurs, it involves only a small number of fibroblasts relative to the number seeded.

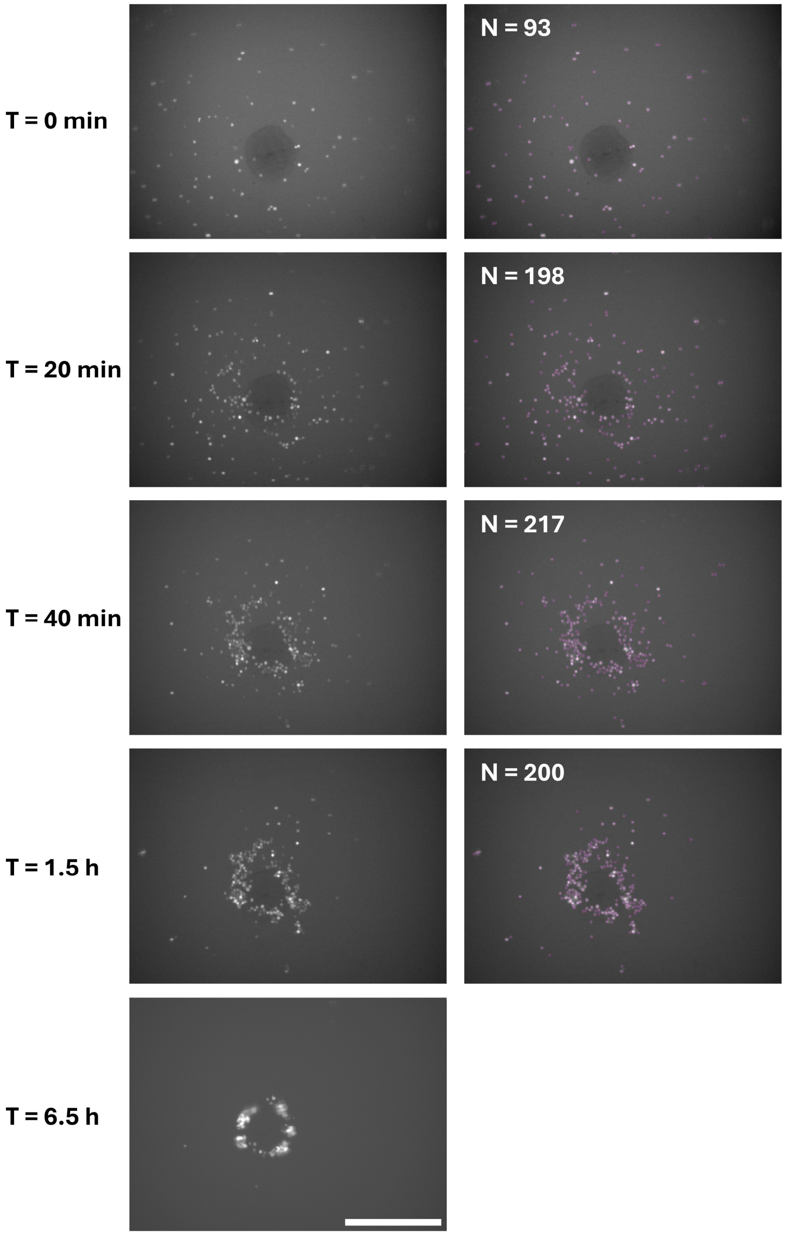

Fig. S11 | Fibroblast detection over time during early cluster formation. Left column: green-channel TP images; Right column: fibroblasts segmented using TrackMate. Numbers indicate detected fibroblast counts. Scale bars: 500 μm.

13. Wound healing assay

To identify compounds that modulate fibroblast motility and could influence spheroid-fibroblast interactions, we performed a preliminary drug screen focusing on compounds previously reported to inhibit cell migration or reduce cell mobility. To evaluate their impact on fibroblast behavior, we conducted a two-dimensional wound healing assay using 3T3 cells (Fig. S12). 3T3 cells were seeded at a density of $1 \cdot 10^4$ cells per well in collagen-coated 96-well plates (Collagen Type I, rat tail, Sigma Aldrich, St. Louis, MO, USA) and were allowed 72 hours to form a confluent monolayer. Wounds were made using a 0.1-10 µl pipette tip, and drugs were added after 1 hour at four different concentrations: 0.01, 0.005, 0.001 and 0.0005 mg/ml. Wells were imaged every 30 minutes for 24 hours. This assay enabled efficient quantification of cell migration in response to drug treatment and served as a rapid pre-screening tool to identify candidates that significantly impair fibroblast mobility.

From an initial panel of compounds, we selected four drugs that demonstrated measurable effects on 3T3 motility and compared them to untreated (NT) and DMSO-treated controls; effective treatments inhibited cell migration and maintained the wound gap, whereas ineffective treatments did not restrict motility and the wound closed over time (Fig. S12). These drugs were then advanced to our three-dimensional co-culture spheroid experiments, where their potential to modulate fibroblast-spheroid interactions could be further assessed (Supplementary Note 14).

The selected drugs were 17-N-allylamino-17-demethoxygeldanamycin (17-AAG), nintedanib, ponatinib and infigratinib. 17-AAG is an Hsp90 inhibitor, primarily studied for its anti-cancer properties[13]. Its effects on fibroblasts have also been explored, showing reduced migration distance and impaired directional movement toward wound sites[14]. Nintedanib is a small molecule tyrosine kinase inhibitor approved for the treatment of lung fibrosis. One if its therapeutic mechanisms involves inhibiting fibroblast migration and invasion[15,16]. Ponatinib and infigratinib are both kinase inhibitors used in targeted cancer therapy[17,18]. While primarily designed to block oncogenic signaling, they could potentially impact cellular processes that include cell mobility[19–22].

In addition to these four drugs, we included two additional drugs as negative and positive controls. Staurosporine, a multi-kinase inhibitor known to induce apoptosis in various cell lines[23] was used as a positive control, and enzalutamide, an androgen receptor (AR) inhibitor used primarily to treat prostate cancer[24] was used as a negative control, as 3T3 cells have negligible AR expression.

Based on the 2D assay results, we selected the second and fourth concentrations ($5 \cdot 10^{-3}$ and $5 \cdot 10^{-4}$ mg/ml) for subsequent 3D experiments. This range provided effective inhibition while minimizing cytotoxicity observed at the highest concentration for some of the drugs.

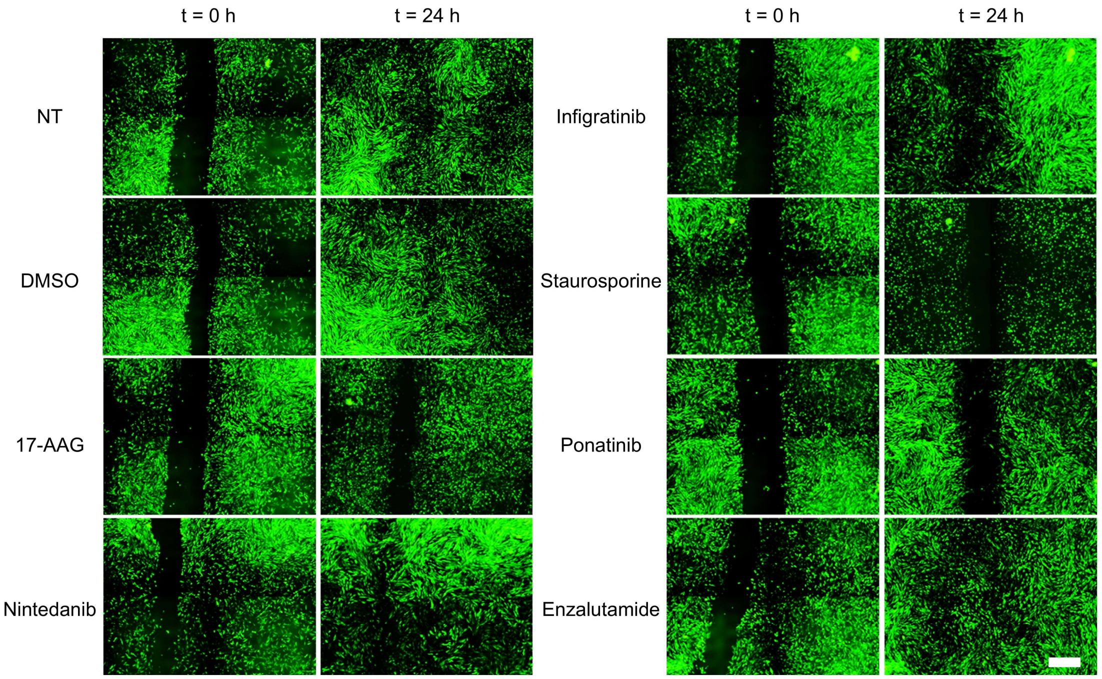

Fig. S12 | In-vitro wound healing assay of 3T3 fibroblasts cells. Representative fluorescence images of the scratch areas after wounding (t = 0 h) and after 24 h treated with different drugs at a concentration of $5 \cdot 10^{-4}$ mg/ml, Green represents GFP signal from 3T3 cells. Scale bar: 500 μm.

14. Drug effect on spheroid-fibroblast interaction

Following the 2D wound healing experiment (Supplementary Note 13), we proceeded our 3D imaging experiments with three drugs that showed inhibitory effects on fibroblast motility (17-AAG, infigratinib and ponatinib), alongside staurosporine and enzalutamide as positive and negative controls, respectively. Nintedanib was excluded due to its relatively low impact on fibroblast motility and its intrinsic fluorescence under our imaging conditions.

To evaluate the impact of the selected compounds on 3D fibroblast-tumor spheroid interactions, we added the selected drugs to spheroid-fibroblast samples seeded in a 96 round-bottom well plate, and imaged the samples using our system, enabling simultaneous assessment of multiple drugs and concentrations under identical conditions. To form spheroid-fibroblast samples, FaDu cells (1000 per well) were seeded and allowed to form spheroids for 24 hours, after which GFP-expressing 3T3 fibroblasts (100 per well, 1:10 ratio) were added. Following a 1-hour incubation to allow fibroblasts to settle, drugs were added at 4 concentrations $5 \cdot 10^{-3}$, $5 \cdot 10^{-4}$, $3.7 \cdot 10^{-6}$ and $3.7 \cdot 10^{-7}$ mg/ml. Samples were imaged once an hour for several days using our PSF-engineered high-throughput microscope.

Representative green fluorescence images show the 3T3 cluster formation under each drug condition after 24 hours (Fig. S13). The acquired images revealed varying drug effects on the characteristic fibroblast cluster pattern. Among all conditions, ponatinib had the strongest concentration-dependent effects on fibroblast cluster patterns. These results motivated further investigation of ponatinib effect on the interactions in 3D, where we focused on the two highest concentrations as these showed the strongest effect on the interactions (Fig. 6).

For sample characterization over time (Fig. 6), spheroid diameters and cluster z-depths were measured at each time point for samples under low drug concentration (low), high drug concentration (high) and untreated (NT) conditions, respectively. Spheroid diameters were estimated by segmenting spheroids in images acquired in the bright-field channel. Segmentation was performed on the Laplacian of the images, for enhanced contrast, and executed using OpenCV[1] functions *connectedComponents* and *threshold*. Diameters were calculated by estimating the spheroid surface area from the pixel count of segmented spheroids, assuming spheroids are circular.

Cluster z-depths were calculated according to the steps described in Supplementary Note 4, which include 2D localization of clusters or cells, their cropping in two z-planes and 3D localization using the DfD algorithm. Localizations with a correlation score lower than 0.85 were excluded from the analysis.

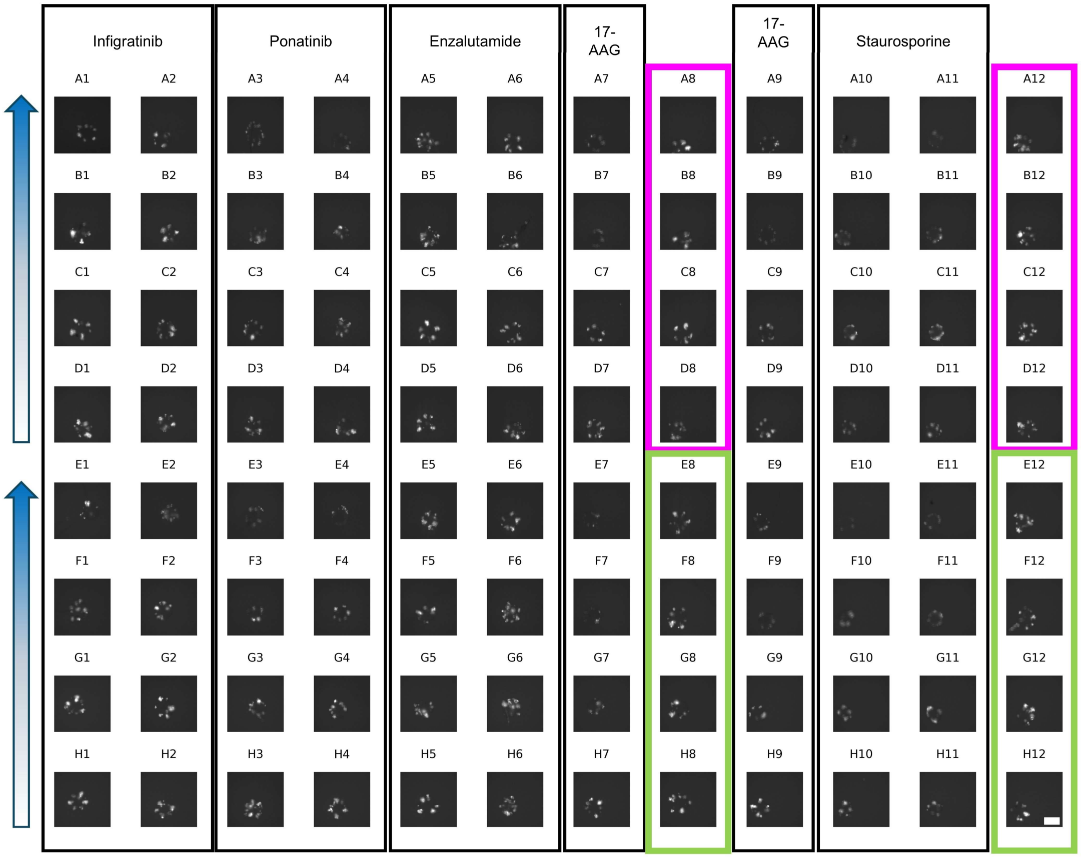

Fig. S13 | Fibroblast interactions with FaDu spheroids under drug influence. The interactions were examined using the green fluorescence channel 24 hours after drug addition. Each column represents a specific drug treatment. Rows correspond to drug concentrations, with the highest concentration ($5 \cdot 10^{-3}$ mg/ml) in rows A and E, and the lowest concentration ($3.7 \cdot 10^{-7}$ mg/ml) in rows D and H, as indicated by the blue arrows. Wells outlined in pink indicate DMSO-treated controls, and wells outlined in green represent untreated (NT) controls. White signal indicates 3T3 fluorescence, FaDu cells are not fluorescent. Scale bar: 300 μm.

15. FaDu and 3T3 viability assays under ponatinib treatment

Our 3D imaging analysis for spheroid-fibroblast samples under ponatinib treatment demonstrated the drug effect on 3T3 mobility (Fig. S13 and Fig. 6). Here, we further evaluate the effect of ponatinib on 3T3 viability, providing a complementary assessment of the drug effect on the cells (Fig. S14). To assess viability, we seeded 1,000 FaDu or 100 3T3 cells per well and added ponatinib at four concentrations. Viability was quantified using Promega® (Madison, WI, USA) CellTiter-Glo® (CTG) 3D kit, according to the manufacturer's instructions.

Fig. S14 shows viability loss at the two highest concentrations. In our 3D analysis (Fig. 6), we characterized the interactions over the first 35 hours under drug influence at these two concentrations, thus we tracked the process of viability loss at its early stages.

Specifically, the highest concentration resulted in cell death in both cell lines. At the second highest concentration, FaDu spheroids were less affected, while 3T3 clusters retained only 40% viability after 24 hours. Notably, ponatinib also affected the morphology of FaDu spheroids. At the highest concentration an expansion of the spheroid was observed at 24 hours, following its disassembly, while at the second-highest concentration, spheroid growth was inhibited compared to untreated spheroids. These effects are also visible in the spheroid diameter analysis presented in Fig. 6.

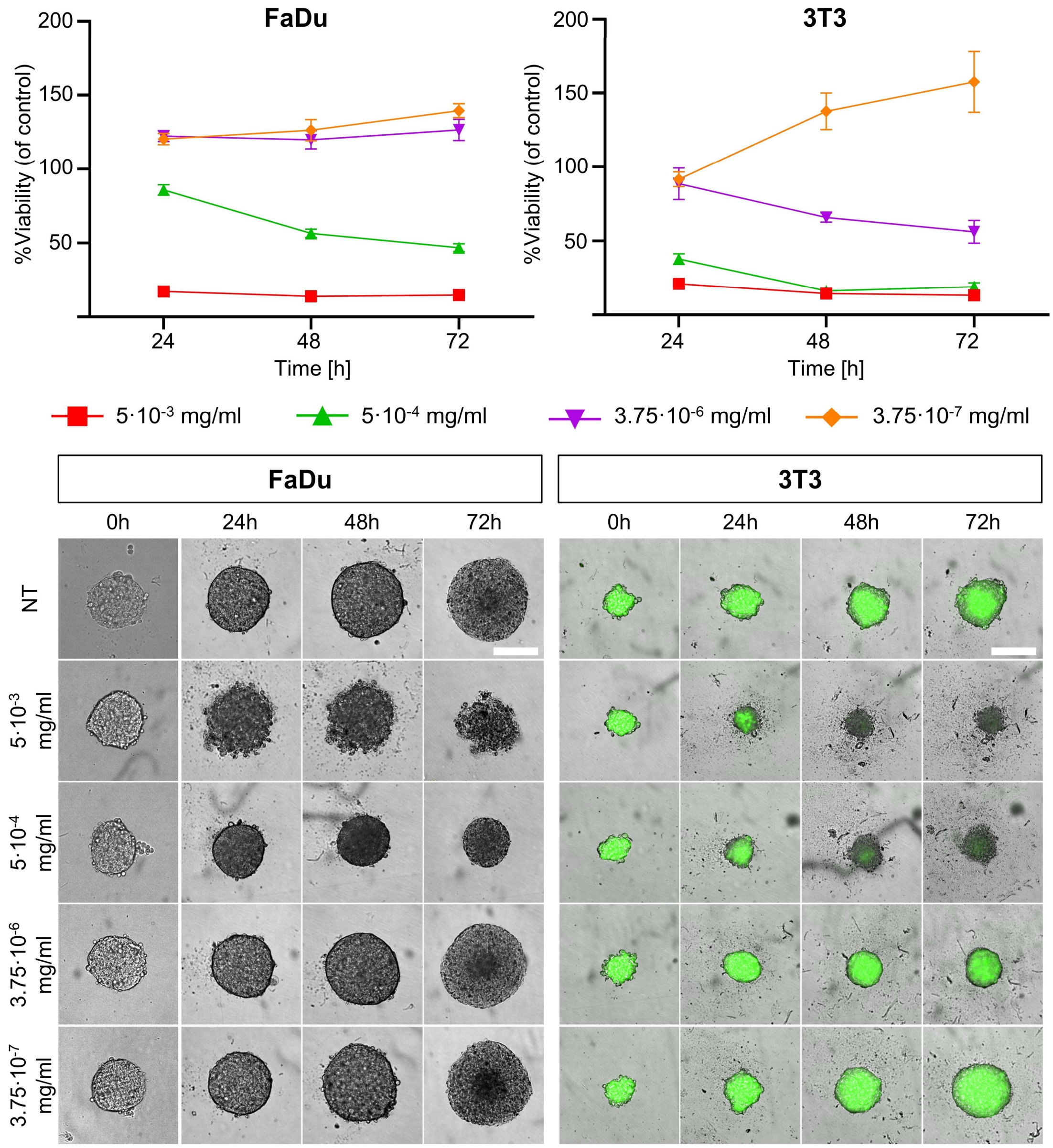

Fig. S14 | Cell viability assay for FaDu and 3T3 3D cell cultures. 3D cell cultures of FaDu (left) and 3T3 (right) cells were incubated with 4 concentrations of ponatinib for 24, 48 and 72 hours. Top - CTG 3D results. Experiments were conducted with three replicates per condition. Statistical analysis was performed using GraphPad Prism (GraphPad 10 Software). In order to standardize the results, each well was normalized relative to the control. Error bars depict the standard deviation (SD). Bottom - representative bright-field and fluorescence (GFP) images for FaDu and 3T3 clusters, respectively, acquired using IncuCyte S3. Green represents GFP signal from 3T3 cells. Scale bars: 200 µm.

References

1. Bradski, G. The opencv library. *Dr Dobbs J. Softw. Tools Prof. Program.* **25**, 120–123 (2000).
2. Jena, P. V. *et al.* Photoluminescent carbon nanotubes interrogate the permeability of multicellular tumor spheroids. *Carbon* **97**, 99–109 (2016).
3. Diaspro, A., Federici, F. & Robello, M. Influence of refractive-index mismatch in high-resolution three-dimensional confocal microscopy. *Appl. Opt.* **41**, 685 (2002).
4. Hell, S., Reiner, G., Cremer, C. & Stelzer, E. H. K. Aberrations in confocal fluorescence microscopy induced by mismatches in refractive index. *J. Microsc.* **169**, 391–405 (1993).
5. Petrov, P. N. & Moerner, W. E. Addressing systematic errors in axial distance measurements in single-emitter localization microscopy. *Opt. Express* **28**, 18616 (2020).
6. Opatovski, N. *et al.* Depth-enhanced high-throughput microscopy by compact PSF engineering. *Nat. Commun.* **15**, 4861 (2024).
7. Otsu, N. A Threshold Selection Method from Gray-Level Histograms. *IEEE Trans. Syst. Man Cybern.* **9**, 62–66 (1979).
8. P. Thevenaz, U.E. Ruttimann, M. Unser. A Pyramid Approach to Subpixel Registration Based on Intensity. *IEEE Trans. Image Process.* **7**, 27–41 (1998).
9. Van Der Walt, S. *et al.* scikit-image: image processing in Python. *PeerJ* **2**, e453 (2014).
10. Avrashami, M., Niezni, D., Meron Azagury, D., Sason, H. & Shamay, Y. Green/red fluorescent protein disrupting drugs for real-time permeability tracking in three-dimensional tumor spheroids. *Bioeng. Transl. Med.* **10**, e10731 (2024).
11. Tinevez, J.-Y. *et al.* TrackMate: An open and extensible platform for single-particle tracking. *Methods* **115**, 80–90 (2017).
12. Ershov, D. *et al.* TrackMate 7: integrating state-of-the-art segmentation algorithms into tracking pipelines. *Nat. Methods* **19**, 829–832 (2022).
13. Dimopoulos, M.-A., Mitsiades, C. S., Anderson, K. C. & Richardson, P. G. Tanespimycin as Antitumor Therapy. *Clin. Lymphoma Myeloma Leuk.* **11**, 17–22 (2011).
14. Yun, I. S. *et al.* Heat Shock Protein 90 Inhibitor (17-AAG) Induces Apoptosis and Decreases Cell Migration/Motility of Keloid Fibroblasts. *Plast. Reconstr. Surg.* **136**, 44e (2015).
15. Habiel, D., Espindola, M., Narayanan, R., Jones, I. & Lucia Coelho, A. Modulation of Normal and Idiopathic Pulmonary Fibrosis Lung Fibroblast Motility and Invasion by Nintedanib | A74. REGULATORY MECHANISMS OF THE MOLECULAR PATHWAYS IN FIBROSIS. *Am. Thorac. Soc. Int. Conf. Meet. Abstr. Am. Thorac. Soc. Int. Conf. Meet. Abstr.* **195**, Abstract A2477 (2017).
16. Lin, X. *et al.* Nintedanib inhibits TGF-β-induced myofibroblast transdifferentiation in human Tenon's fibroblasts. *Mol. Vis.* **24**, 789–800 (2018).

17. Tan, F. H., Putoczki, T. L., Stylli, S. S. & Luwor, R. B. Ponatinib: a novel multi-tyrosine kinase inhibitor against human malignancies. *OncoTargets Ther.* **12**, 635–645 (2019).
18. Yu, J., Mahipal, A. & Kim, R. Targeted Therapy for Advanced or Metastatic Cholangiocarcinoma: Focus on the Clinical Potential of Infigratinib. *OncoTargets Ther.* **14**, 5145–5160 (2021).
19. Gao, Y., Ding, Y., Tai, X., Zhang, C. & Wang, D. Ponatinib: An update on its drug targets, therapeutic potential and safety. *Biochim. Biophys. Acta BBA - Rev. Cancer* **1878**, 188949 (2023).
20. Sadovnik, I. *et al.* Identification of Ponatinib as a potent inhibitor of growth, migration and activation of neoplastic eosinophils carrying FIP1L1-PDGFRA. *Exp. Hematol.* **42**, 282-293.e4 (2014).
21. Fu, Y. *et al.* Infigratinib, a Selective Fibroblast Growth Factor Receptor Inhibitor, Suppresses Stent-Induced Tissue Hyperplasia in a Rat Esophageal Model. *Cardiovasc. Intervent. Radiol.* **46**, 1267–1275 (2023).
22. Huynh, H. *et al.* Infigratinib Mediates Vascular Normalization, Impairs Metastasis, and Improves Chemotherapy in Hepatocellular Carcinoma. *Hepatology* **69**, 943 (2019).
23. Bertrand, R., Solary, E., O'Connor, P., Kohn, K. W. & Pommier, Y. Induction of a Common Pathway of Apoptosis by Staurosporine. *Exp. Cell Res.* **211**, 314–321 (1994).
24. Hoffman-Censits, J. & Kelly, Wm. K. Enzalutamide: A Novel Antiandrogen for Patients with Castrate-Resistant Prostate Cancer. *Clin. Cancer Res.* **19**, 1335–1339 (2013).
