# Supplementary material for: Symmetric cancer spheroid-fibroblast organization revealed in 3D by high-throughput microscopy
Source: Commun Biol. 2026 Jul 27;9:1023. doi: 10.1038/s42003-026-10592-3 (PMC13415527; doi:10.1038/s42003-026-10592-3)
Supplement: Supplementary file 2 — Description of Additional Supplementary Files [file 42003_2026_10592_MOESM2_ESM.docx]

**Description of Additional Supplementary Files**

**File name:** Supplementary Data 1

**Description:** Source data for the graphs presented in this paper.

**File name:** Supplementary Video 1

**Description:** Single cell interaction with a spheroid over time. Timelapse imaging of single fibroblasts interacting with a spheroid (showing the same example as in Fig. 3), presented as a composite of bright-field and TP green-channel images over time. Between 14 and 20 hours, a single fibroblast, highlighted by a white box, is visibly moving along the spheroid surface, near its axial center. Scale bar: 100 µm.

**File name:** Supplementary Video 2

**Description:** Spheroid-fibroblast interaction over time. Timelapse imaging of fibroblasts interacting with a spheroid, presented as a composite of bright-field and TP green-channel images over time, and showing fibroblast cluster formations. Between 25 and 31 hours, two smaller fibroblast clusters, highlighted by white boxes, are seen migrating along the spheroid surface near its axial center, moving towards the closest spheroid-bound cluster, with which they merge to maintain a symmetrical spheroid-fibroblast structure. Scale bar: 200 µm.
