# Supplementary material for: Symmetric cancer spheroid-fibroblast organization revealed in 3D by high-throughput microscopy
Source: Commun Biol. 2026 Jul 27;9:1023. doi: 10.1038/s42003-026-10592-3 (PMC13415527; doi:10.1038/s42003-026-10592-3)
Supplement: Supplementary file 6 — Reporting Summary [file 42003_2026_10592_MOESM6_ESM.pdf]

nature portfolio

Corresponding author(s): Yoav Shechtman, Yosi Shamay

Last updated by author(s): Jun 7, 2026

Reporting Summary

Nature Portfolio wishes to improve the reproducibility of the work that we publish. This form provides structure for consistency and transparency in reporting. For further information on Nature Portfolio policies, see our Editorial Policies and the Editorial Policy Checklist.

Statistics

For all statistical analyses, confirm that the following items are present in the figure legend, table legend, main text, or Methods section.

n/a	Confirmed	
☐	☒	The exact sample size (*n*) for each experimental group/condition, given as a discrete number and unit of measurement
☐	☒	A statement on whether measurements were taken from distinct samples or whether the same sample was measured repeatedly
☐	☒	The statistical test(s) used AND whether they are one- or two-sided<br>*Only common tests should be described solely by name; describe more complex techniques in the Methods section.*
☐	☒	A description of all covariates tested
☒	☐	A description of any assumptions or corrections, such as tests of normality and adjustment for multiple comparisons
☐	☒	A full description of the statistical parameters including central tendency (e.g. means) or other basic estimates (e.g. regression coefficient) AND variation (e.g. standard deviation) or associated estimates of uncertainty (e.g. confidence intervals)
☒	☐	For null hypothesis testing, the test statistic (e.g. *F*, *t*, *r*) with confidence intervals, effect sizes, degrees of freedom and *P* value noted<br>*Give P values as exact values whenever suitable.*
☒	☐	For Bayesian analysis, information on the choice of priors and Markov chain Monte Carlo settings
☒	☐	For hierarchical and complex designs, identification of the appropriate level for tests and full reporting of outcomes
☒	☐	Estimates of effect sizes (e.g. Cohen's *d*, Pearson's *r*), indicating how they were calculated

Our web collection on statistics for biologists contains articles on many of the points above.

Software and code

Policy information about availability of computer code

Data collection	Data was acquired using custom acquisition software for Incucyte® S3 Live-Cell Analysis System (Sartorius).
Data analysis	Data analysis was done using Python and Matlab R2021b. Code is publicly available at https://github.com/NoamZoref/HTM-LocZ. The version of the code described in this paper is archived at https://doi.org/10.5281/zenodo.20394791

For manuscripts utilizing custom algorithms or software that are central to the research but not yet described in published literature, software must be made available to editors and reviewers. We strongly encourage code deposition in a community repository (e.g. GitHub). See the Nature Portfolio guidelines for submitting code & software for further information.

Data

Policy information about availability of data

All manuscripts must include a data availability statement. This statement should provide the following information, where applicable:

- Accession codes, unique identifiers, or web links for publicly available datasets
- A description of any restrictions on data availability
- For clinical datasets or third party data, please ensure that the statement adheres to our policy

The source data for the graphs presented in this paper are available in Supplementary Data 1. All other data are available from the corresponding author upon reasonable request.

Research involving human participants, their data, or biological material

Policy information about studies with human participants or human data. See also policy information about sex, gender (identity/presentation), and sexual orientation and race, ethnicity and racism.

Reporting on sex and gender	n/a
Reporting on race, ethnicity, or other socially relevant groupings	n/a
Population characteristics	n/a
Recruitment	n/a
Ethics oversight	n/a

Note that full information on the approval of the study protocol must also be provided in the manuscript.

Field-specific reporting

Please select the one below that is the best fit for your research. If you are not sure, read the appropriate sections before making your selection.

☒ Life sciences ☐ Behavioural & social sciences ☐ Ecological, evolutionary & environmental sciences

For a reference copy of the document with all sections, see nature.com/documents/nr-reporting-summary-flat.pdf

Life sciences study design

All studies must disclose on these points even when the disclosure is negative.

Sample size	Sample sizes were chosen based on the high-throughput imaging capabilities of the Incucyte® S3 Live-Cell Analysis System (Sartorius). Samples were examined in 96-well plates, each containing 96 individual spheroid samples, yielding multiple replicates per experiment.
Data exclusions	Samples with visible artifacts (e.g., dust and fibers) were excluded from analysis. For the analysis of spheroid-fibroblast interaction patterns, samples for which clusters could not be visually separated were excluded from the analysis of number of formed clusters vs. the number of FaDu cells. In addition, samples for which adjacent clusters could not be resolved by computational segmentation were excluded from the analysis of 3D distances between adjacent clusters for different numbers of FaDu cells. For the analysis of drug effect on spheroid-fibroblast interactions, cluster localizations with low localization scores were excluded from the analysis.
Replication	In our high-throughput experiments, replicates were generated for each examined condition. All attempts at replication of the experiments were successful.
Randomization	Samples were allocated into experimental groups by biological design. Controlled parameters include the number of seeded cancer cells, the ratio of seeded fibroblast cells, ponatinib drug concentration and cancer cell type.
Blinding	The investigators were not blinded to group allocation during data collection and analysis.

Reporting for specific materials, systems and methods

We require information from authors about some types of materials, experimental systems and methods used in many studies. Here, indicate whether each material, system or method listed is relevant to your study. If you are not sure if a list item applies to your research, read the appropriate section before selecting a response.

Materials & experimental systems

n/a	Involved in the study	
☐	☒	Antibodies
☐	☒	Eukaryotic cell lines
☒	☐	Palaeontology and archaeology
☒	☐	Animals and other organisms
☒	☐	Clinical data
☒	☐	Dual use research of concern
☒	☐	Plants

Methods

n/a	Involved in the study	
☒	☐	ChIP-seq
☒	☐	Flow cytometry
☒	☐	MRI-based neuroimaging

Antibodies

Antibodies used	Primary antibodies: anti-vimentin, ab24525, polyclonal antibody (Chk pAb), LOT 1091824-14, Zotal;<br>anti-Nectin-4/PVRL4, MABT64, clone N4.61, LOT 3744860, Sigma-Aldrich.<br>Secondary antibodies: anti-chicken Alexa Fluor® 594, ab150176, Goat pAb to Chk IgY, LOT GR3401044-2, Zotal;<br>anti-mouse Alexa Fluor® 488, ab150117, Goat pAb to Ms IgG, LOT GR3398339-4, Zotal.
Validation	The anti-Nectin-4/PVRL4 antibody has been validated for the detection of human Nectin-4 and is used in immunohistochemical applications. Nectin-4 is highly expressed in many epithelial cancers, including head and neck squamous cell carcinomas, such as the FaDu cell line. Human Protein Atlas data indicate that the FaDu cell line expresses Nectin-4 at a relatively high level (13.8 normalized transcript per million (nTPM); expression across cell lines reaches approximately 60 nTPM). Therefore, the antibody was expected to specifically label FaDu epithelial cancer cells of human origin, while showing no reactivity toward murine 3T3 fibroblasts.<br>The anti-vimentin antibody has been validated by the manufacturer for immunocytochemistry/immunofluorescence (ICC/IF) applications in human, mouse, and rat samples. Vimentin is a well-established mesenchymal marker that is strongly expressed in fibroblasts, while FaDu cells exhibit low expression according to Human Protein Atlas data (2.7 nTPM), placing them among the lower-expressing cell lines in the dataset, where expression levels ranged up to approximately 14,000 nTPM. It was expected to strongly label 3T3 fibroblasts and potentially a subset of FaDu cells exhibiting mesenchymal characteristics.

Eukaryotic cell lines

Policy information about cell lines and Sex and Gender in Research

Cell line source(s)	The SK-136 cell line originated from murine hepatoblastoma cells; the FaDu cell line originated from a human hypopharyngeal squamous cell carcinoma in a male; the 3T3-ZsGreen expressing fibroblast cell line originated from mouse embryonic fibroblasts; the Cal33 cell line originated from a human tongue squamous cell carcinoma in a male.
Authentication	Cell lines were not independently authenticated by the authors. The cell lines were obtained from reputable sources, and cells were used according to the suppliers' guidelines.
Mycoplasma contamination	The cell lines were not tested for mycoplasma contamination.
Commonly misidentified lines<br>(See ICLAC register)	n/a

Plants

Seed stocks	n/a
Novel plant genotypes	n/a
Authentication	n/a
